# Proteolysis of the low density lipoprotein receptor by bone morphogenetic protein-1 regulates cellular cholesterol uptake

**DOI:** 10.1038/s41598-019-47814-0

**Published:** 2019-08-06

**Authors:** Sreemoti Banerjee, Robert J. Andrew, Christopher J. Duff, Kate Fisher, Carolyn D. Jackson, Catherine B. Lawrence, Nobuyo Maeda, Daniel S. Greenspan, Katherine A. B. Kellett, Nigel M. Hooper

**Affiliations:** 1 School of Biological Sciences, Faculty of Biology, Medicine and Health, University of Manchester, Manchester Academic Health Sciences, Manchester, M13 9PT UK; 2 School of Molecular and Cellular Biology, Faculty of Biological Sciences, University of Leeds, Leeds, LS2 9JT UK; 3 Department of Pathology and Laboratory Medicine, University of North Carolina, Chapel Hill, North Carolina USA; 4 Department of Cell and Regenerative Biology, School of Medicine and Public Health, University of Wisconsin, Madison, WI USA; 5 Present Address: Jack Birch Unit for Molecular Carcinogenesis, Department of Biology, University of York, York, YO10 5DD UK; 6 Present Address: Department of Neurobiology, The University of Chicago, Chicago, IL 60637 USA; 7 Present Address: Department of Clinical Biochemistry, University Hospitals of North Midlands NHS Trust, Stoke-on-Trent, ST4 6QG UK

**Keywords:** Mechanisms of disease, Dyslipidaemias

## Abstract

The development of cardiovascular disease is intimately linked to elevated levels of low-density lipoprotein (LDL) cholesterol in the blood. Hepatic LDL receptor (LDLR) levels regulate the amount of plasma LDL. We identified the secreted zinc metalloproteinase, bone morphogenetic protein 1 (BMP1), as responsible for the cleavage of human LDLR within its extracellular ligand-binding repeats at Gly_171_↓Asp_172_. The resulting 120 kDa membrane-bound C-terminal fragment (CTF) of LDLR had reduced capacity to bind LDL and when expressed in LDLR null cells had compromised LDL uptake as compared to the full length receptor. Pharmacological inhibition of BMP1 or siRNA-mediated knockdown prevented the generation of the 120 kDa CTF and resulted in an increase in LDL uptake into cells. The 120 kDa CTF was detected in the livers from humans and mice expressing human LDLR. Collectively, these results identify that BMP1 regulates cellular LDL uptake and may provide a target to modulate plasma LDL cholesterol.

## Introduction

Plasma low-density lipoprotein (LDL) cholesterol is a major determinant of cardiovascular disease risk and recent meta-analysis of genetic and clinical evidence unequivocally established that raised LDL cholesterol, hypercholesterolaemia, causes atherosclerotic cardiovascular disease^1,2^. Current medications (such as statins, bile acid sequestrants, fibrates and cholesterol absorption inhibitors) are effective at reducing plasma LDL cholesterol in many people^3^. However, a considerable number of individuals fail to reach target plasma LDL cholesterol levels with these medications, while others cannot tolerate the side effects^4^. Thus, there is the ongoing need for a better understanding of the biological mechanisms controlling plasma LDL cholesterol in order to identify alternative therapeutic strategies for hypercholesterolaemia.

The number of LDL receptors (LDLR) expressed on the surface of hepatocytes is the primary determinant of plasma LDL levels^1^. The mature human LDLR is a single chain transmembrane glycoprotein of 839 amino acids consisting of a multi-domain structure. The N-terminal 292 amino acids of the extracellular region form the ligand binding domain which contains seven cysteine rich repeat sequences, each 40 amino acids in length, known as LDLR type A (LA) repeats, which are connected by small linker regions. LDLR binds plasma LDL and the complex is then internalized via clathrin-mediated endocytosis. In the acidic environment of the endosome, the LDL particle dissociates from the receptor, which is then recycled back to the cell surface, while the cholesterol is released into the cell^1^.

Post-translational regulation of LDLR controls its ability to bind and endocytose LDL. Inducible degrader of LDLR (IDOL), an E3 ubiquitin ligase, ubiquitinates the cytoplasmic tail of LDLR and targets it for lysosomal degradation^5,6^. Proprotein convertase subtilisin kexin type 9 (PCSK9) interacts with LDLR to reroute it from the cell surface recycling pathway toward late endocytic compartments for degradation^7,8^, and disruption of the PCSK9-LDLR interaction has emerged as a therapeutic strategy for hypercholesterolaemia^9^. In addition, LDLR is subject to targeted proteolytic cleavage. For example, LDLR was reported recently to undergo γ-secretase-mediated cleavage which in turn induces LDLR lysosomal degradation^10^ and the action of an unidentified protease cleaving LDLR within its LA repeats produced a 120 kDa C-terminal fragment (CTF)^11,12^. Further understanding of these proteolytic mechanisms and the role they play could provide additional therapeutic targets for the treatment of hypercholesterolaemia.

Here, through an *in silico* bioinformatics approach validated by studies with recombinant proteins, cellular models and human and animal tissues, we show for the first time that the secreted zinc metalloproteinase, bone morphogenetic protein 1 (BMP1; also known as procollagen C-peptidase), proteolytically cleaves human LDLR between the fourth and fifth ligand binding repeats at the Gly_171_↓Asp_172_ peptide bond. Cleavage of LDLR by BMP1 reduced the binding of LDL and regulated the cellular uptake of LDL.

## Results

### Human LDLR is proteolytically cleaved in its extracellular ligand binding domain

**Figure 1:**
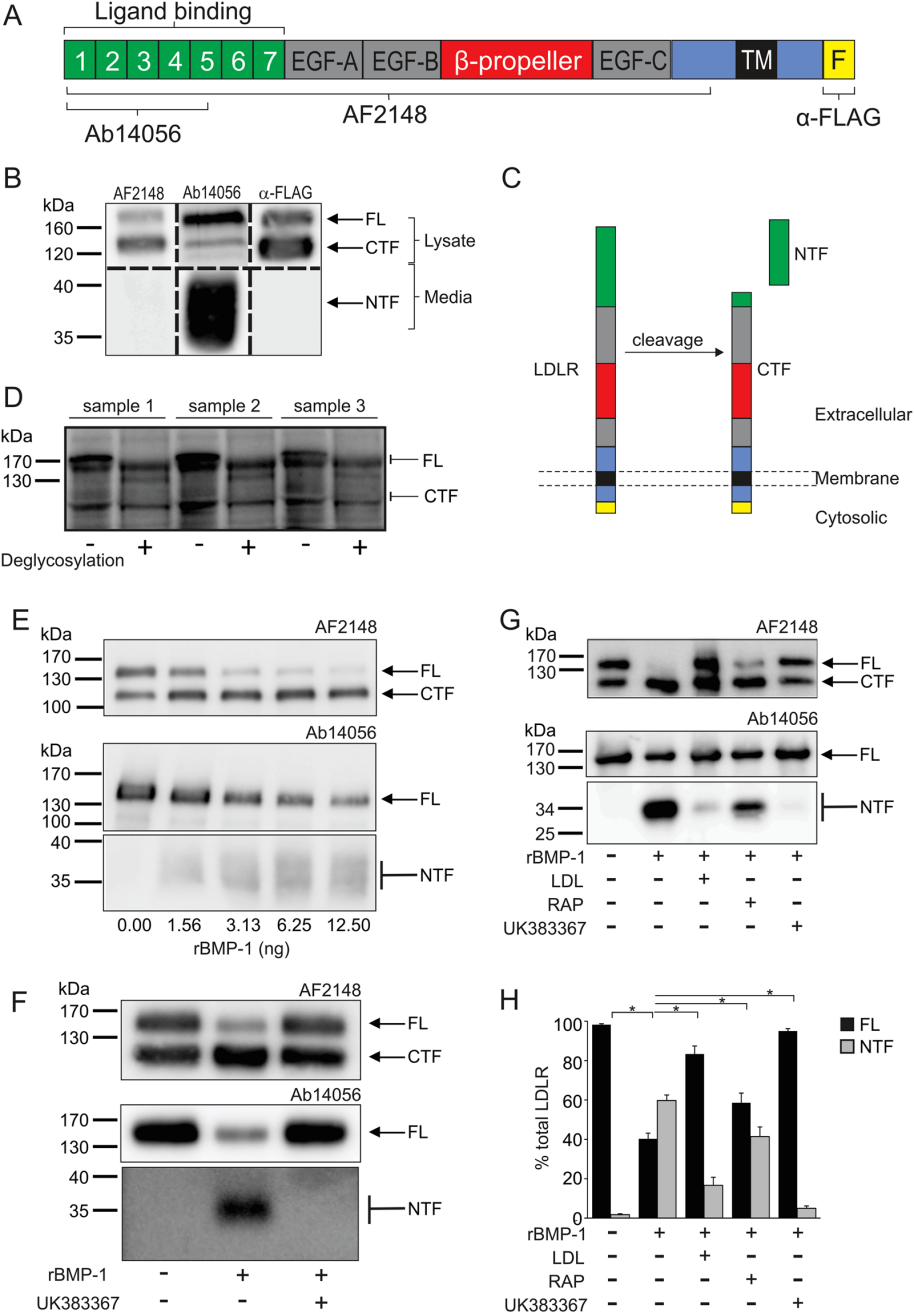
Human LDLR is proteolytically cleaved in its extracellular ligand binding domain by BMP1. (**A**) Schematic of the domain organisation of LDLR with a C-terminal FLAG tag showing the epitopes detected by the antibodies used in the study, antibody AF2148 (R&D Systems) raised against the entire ectodomain of LDLR, antibody Ab14056 (Abcam) raised against a recombinant protein fragment corresponding to amino acids 29–205 of LDLR) and antibody α-FLAG (Sigma-Aldrich) the anti-FLAG M2 antibody. TM, transmembrane domain; EGF, epidermal growth factor-like domain; F, FLAG epitope. (**B**) Immunoblot analysis with the indicated antibody of lysates and conditioned media samples from HepG2 cells expressing full-length FLAG-tagged human LDLR. Bands of interest were cropped from western blots of either media or lysate samples using each of the three antibodies. Images from separate western blots were combined but are separated by the dashed black line. Full blot images are presented in the Supplementary western blot dataset. (**C**) Schematic showing the proposed cleavage of the 160 kDa full-length (FL) LDLR to generate the 36–40 kDa NTF and 120 kDa CTF. (**D**) Immunoblot analysis of LDLR (antibody AF2148) without and with deglycosylation in liver biopsy samples from three separate individuals. The blot image was cropped to highlight the FL and CTF bands, full blot images are presented in the Supplementary western blot dataset. (**E**) Immunoblot analysis following incubation of rhLDLR (500 ng) with increasing amounts of rBMP1 at 37 °C for 1 h. (**F**) Immunoblot analysis following incubation of rhLDLR (500 ng) with rBMP1 (12.5 ng) in the absence or presence of the BMP1 inhibitor UK383367 (10 μM) at 37 °C for 1 h. (**G**) Immunoblot analysis following pre-incubation of rhLDLR (500 ng) in the absence or presence of LDL (5 µg), RAP (7.14pmol) or UK383367 (10 μM) for 30 min on ice followed by the addition of 12.5 ng rBMP1 and further incubation at 37 °C for 1 h. (**H**) Densitometric analysis of the Ab14056 immunoblot from (**C**) to determine the amount of FL and NTF as a percentage of total LDLR, data shown as mean ± SEM, statistical analysis using ANOVA with Tukey post-hoc pairwise analysis *p < 0.05, n = 3. For panels E–G, blot images were cropped to highlight the FL and CTF bands using the AF2148 antibody and the FL and NTF bands using the Ab14056 antibody due to different exposure times for visualisation of the FL and NTF bands. Full blot images are presented in the Supplementary western blot dataset.

To investigate the proteolytic cleavage of human LDLR, HepG2 cells expressing LDLR with a C-terminal FLAG tag (LDLR-FLAG) together with epitope-specific antibodies for LDLR were used (Fig. 1A). In cell lysates all three antibodies (AF2148 antibody raised against the entire ectodomain of LDLR, Ab14056 raised against amino acids 29–205 of LDLR, and anti-FLAG antibody) detected both the 160 kDa full-length protein and a 120 kDa fragment (Fig. 1B). In conditioned media, a 36–40 kDa fragment was detected only by the Ab14056 antibody, indicating that this fragment contains the N-terminus but lacks the C-terminus of the full length protein (Fig. 1B). These data suggested that a proteolytic cleavage event occurs in the extracellular ligand binding domain of the full-length 160 kDa LDLR, generating a 36–40 kDa soluble NTF and a 120 kDa CTF that is still membrane bound (Fig. 1C).

To determine the site in LDLR of this proteolytic cleavage, the FLAG-tagged full-length 160 kDa LDLR and the 120 kDa CTF were subjected to N-terminal protein sequence analysis following immunoprecipitation of the proteins from HepG2 cell lysates using anti-FLAG agarose resin. The sequence of the 160 kDa protein corresponded to the N-terminus of full-length mature LDLR (DRCERNEFQCQD). The N-terminal sequence of the 120 kDa CTF (DSSPCSAFEFHC) corresponded to a region in the middle of the ligand-binding domain (Supplementary Fig. S1). The N-terminal sequences of the 160 kDa and 120 kDa proteins present in a commercially sourced (R&D Systems) purified recombinant LDLR preparation were also determined. The N-terminal sequence of the 160 kDa protein corresponded to that of full-length mature LDLR (AVGDRxERNE), while the N-terminal sequence of the 120 kDa protein (DSSPxSAFEF) was identical to that of the 120 kDa isolated from HepG2 cells. Together these findings indicate that proteolytic cleavage of full-length LDLR occurs at (or N-terminal to) the Gly_171_-Asp_172_ peptide bond in the primary sequence …VFQG_171_↓DSSP…, which lies in the linker region between the fourth and fifth LA repeats of the ligand-binding domain (Supplementary Fig. S1). These data confirm that the 120 kDa CTF is a truncated form of LDLR, resulting from the removal of LA repeats 1–4 by the action of an unknown protease, and that the 36–40 kDa NTF observed in the HepG2 cell culture medium is most likely the cleaved LA repeats 1–4 (Fig. 1C).

To ascertain the physiological relevance of this proteolytic cleavage event, LDLR in human liver samples was immunoblotted. Both the full-length 160 kDa LDLR and the 120 kDa CTF were observed in three separate human liver samples (Fig. 1D). Deglycosylation of the liver samples resulted in a downward shift in the molecular weight of both the full-length LDLR and the 120 kDa CTF, with two distinct proteins still being observed (Fig. 1D). The presence of two proteins after deglycosylation indicates that the 120 kDa CTF is a truncated form of the 160 kDa full-length LDLR and not a result of differential glycosylation of the same polypeptide. These data indicate that the proteolytic cleavage within the LA repeats of human LDLR occurs *in vivo* and is not an *in vitro* artefact or a phenomenon of immortalized cell culture systems.

### The zinc metalloprotease BMP1 cleaves the LDLR in its ligand binding domain

To identify proteases capable of cleaving LDLR at the Gly_171_↓Asp_172_ peptide bond, the MEROPS peptidase database (https://www.ebi.ac.uk/merops/) was searched using the peptide sequence spanning the identified cleavage site. This search identified BMP1 as a candidate protease for cleaving LDLR. To determine whether BMP1 could cleave LDLR, rhLDLR was incubated with increasing amounts of rBMP1. rBMP1 dose-dependently converted the 160 kDa full-length LDLR into the 120 kDa CTF and 36–40 kDa NTF (Fig. 1E), and the selective small molecule, active site-directed inhibitor (UK383367) of BMP1^13^ completely prevented this cleavage (Fig. 1F). Following complete digestion of the rhLDLR by rBMP1, the N-terminal sequence of the 120 kDa CTF was confirmed as DSSP by N-terminal sequencing. These data confirm that BMP1 cleaves human LDLR at the Gly_171_↓Asp_172_ peptide bond. The cleavage of LDLR by BMP1 was also investigated in the presence of LDL or receptor-associated protein (RAP) to explore whether binding of either of these ligands to LDLR would inhibit its cleavage. BMP1 mediated cleavage of LDLR was significantly inhibited in the presence of either LDL or RAP (Fig. 1G,H) indicating that binding of these ligands inhibits the proteolytic cleavage by BMP1.

### Cleavage of human LDLR by BMP1 reduces the binding and cellular uptake of LDL

**Figure 2:**
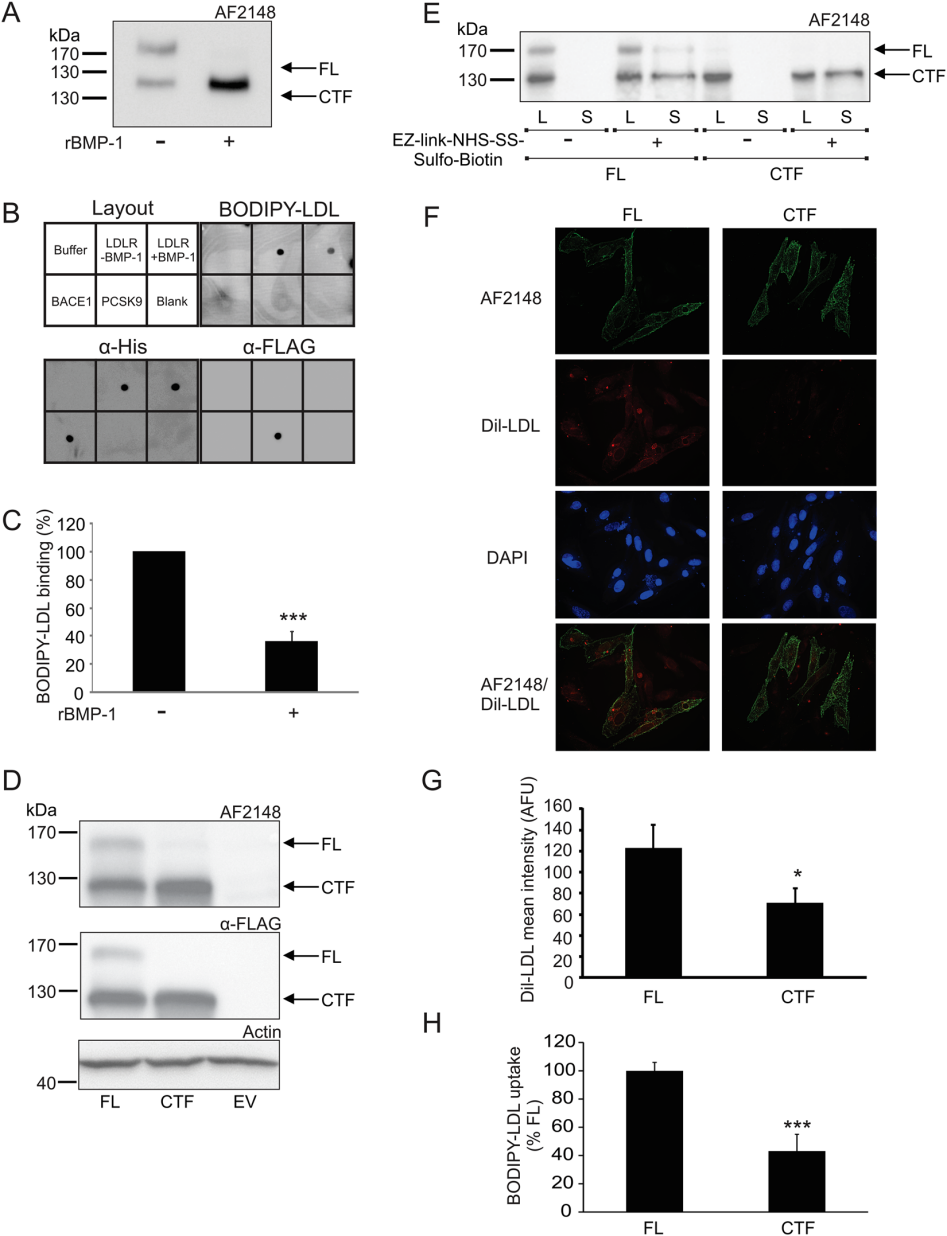
Cleavage of human LDLR by BMP1 reduces the binding and cellular uptake of LDL. (**A**) Immunoblot analysis following incubation of rhLDLR-His (5 μg) with rBMP1 (200 ng) at 37 °C for 1 h. The blot image was cropped to highlight the FL and CTF bands, full blot images are presented in the Supplementary western blot dataset. (**B**) Dot blot of BODIPY^®^-LDL (1 μg/ml) binding to rhLDLR-His digested with BMP1 (from A), BACE1-His and PCSK9-FLAG (all 0.5 μg). Membranes were re-probed with anti-His and anti-FLAG antibodies. (**C**) Densitometric analysis of the BODIPY-LDL dot blot from (**B**) showing the percentage binding to rhLDLR-His in the absence or presence of rBMP1. Data expressed as percentage of control and shown as mean ± SEM, statistical analysis performed using a Student’s T-test ***p < 0.001, n = 4. (**D**) Immunoblot analysis of LDLR with actin as a loading control in lysates from CHO-A7 cells expressing either full-length (FL) LDLR or 120 kDa CTF, or the empty vector (EV). The blot images were cropped to highlight the FL and CTF bands using the AF2148 and anti-FLAG antibodies and actin using the AC15 antibody. The full blot images are presented in the Supplementary western blot dataset. (**E**) Immunoblot analysis of LDLR from lysates (L) and from the cell-surface fraction (S) in CHO-A7 cells expressing either FL LDLR or 120 kDa CTF followed by incubation with either vehicle (DMSO) or EZ-Link™ Sulfo-NHS-SS-Biotin (0.5 mg/ml) for 20 min on ice and immunoprecipitation with Streptavidin-agarose beads for 3 h at 4 °C. The blot image was cropped to highlight the FL and CTF bands using the AF2148 antibody. The full blot image is presented in the Supplementary western blot dataset. (**F**) Immunofluorescence microscopy images showing LDLR expression (green) and Dil-LDL binding (red) in CHO-A7 cells expressing either FL LDLR or 120 kDa CTF followed by incubation with Dil-LDL for 30 s on ice. (**G**) Quantification of Dil-LDL mean intensity in either the FL LDLR or 120 kDa CTF expressing cells from (**F**), data shown as mean ± SEM, statistical analysis performed using a Student’s T-test *p < 0.05, n = 7. (**H**) BODIPY^®^-LDL uptake in CHO-A7 cells expressing either FL LDLR or 120 kDa CTF. Data expressed as percentage of FL following subtraction of empty vector uptake as a baseline and shown as mean ± SEM, statistical analysis performed using a Student’s T-test ***p < 0.001, *p < 0.05, n = 4.

Recombinant full-length LDLR and the CTF generated by digestion with rBMP1 (Fig. 2A) were assessed for their ability to bind BODIPY^®^-LDL using dot blotting. BODIPY^®^-LDL bound to the undigested full-length LDLR, while there was reduced binding to the BMP1 digested CTF (Fig. 2B, top right panel). The BODIPY^®^-LDL did not bind to the control proteins, PCSK9 and BACE1 (Fig. 2B, top panel). The presence of each protein was confirmed by re-probing for the appropriate C-terminal tag (His or FLAG) (Fig. 2B, lower panels). Densitometric analysis of the dot blots indicated that the binding of LDL to the 120 kDa CTF was reduced by 63% compared to the binding to the untreated predominantly full-length 160 kDa LDLR (Fig. 2C), indicating that removal of LA repeats 1–4 of LDLR by BMP1 significantly reduces its ability to bind LDL.

To determine whether the CTF formed by BMP1 cleavage had reduced cellular LDL binding and uptake, cDNAs encoding either full-length LDLR or the 120 kDa CTF were transfected into LDLR-deficient CHO-A7 cells^14^. Both constructs were expressed (Fig. 2D) and both were present on the cell surface as determined by cell surface biotinylation (Fig. 2E). LDL binding (Fig. 2F,G) and uptake (Fig. 2H) were significantly decreased by 42% and 58%, respectively, in cells expressing the CTF compared to those expressing the full-length receptor. Collectively, these data indicate that the 120 kDa CTF generated by proteolytic cleavage of LDLR by BMP1 has a reduced ability to bind and endocytose LDL.

### Genetic knockdown or pharmacological inhibition of BMP1 decreases the proteolytic cleavage of LDLR and increases the cellular uptake of LDL

**Figure 3:**
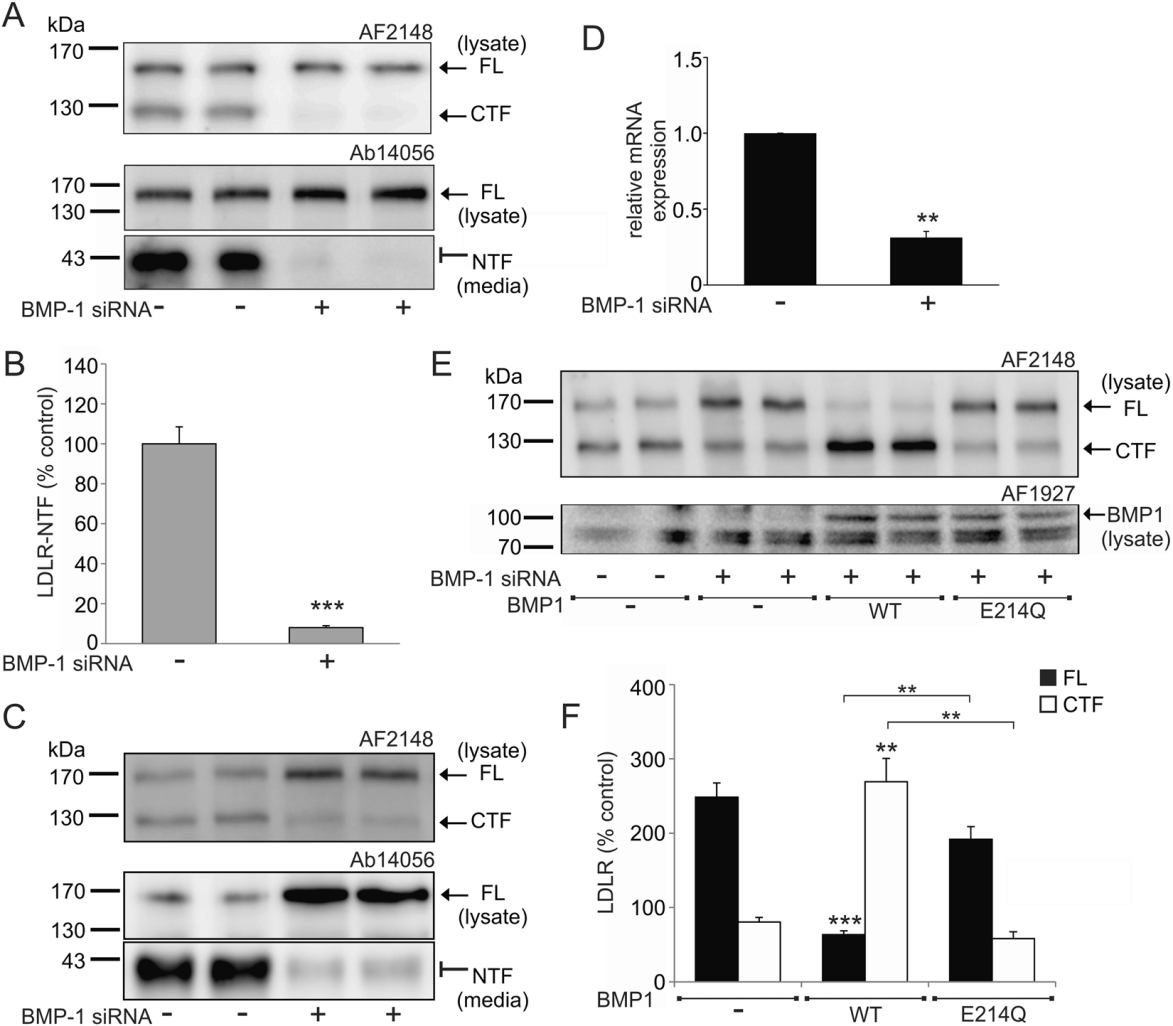
Genetic knockdown of BMP1 decreases the proteolytic cleavage of LDLR. (**A**) Immunoblot analysis of LDLR in cell lysates and conditioned media from HepG2 cells treated either with siRNA against BMP1 (Dharmacon) or with a non-targeting control siRNA. Blot images were cropped to highlight the FL and CTF bands in cell lysates using the AF2148 antibody and the FL and NTF bands in cell lysates and conditioned media, respectively, using the Ab14056 antibody. Full blot images are presented in the Supplementary western blot dataset. (**B**) Densitometric analysis of the NTF from (**A**). Data expressed as percentage of control and shown as mean ± SEM, statistical analysis performed using a Student’s T-test ***p < 0.001, n = 6. (**C**) Immunoblot analysis of LDLR in cell lysates and conditioned media from HepG2 cells treated either with siRNA against BMP1 (Ambion) or with a non-targeting control siRNA. Blot images were cropped to highlight the FL and CTF bands in cell lysates using the AF2148 antibody and the FL and NTF bands in cell lysates and conditioned media, respectively, using the Ab14056 antibody. Full blot images are presented in the Supplementary western blot dataset. (**D**) Relative expression of BMP1 mRNA in HepG2 cells treated with siRNA against BMP1 (Ambion) or with a non-targeting control. Data expressed relative to control and shown as mean ± SEM, statistical analysis performed using an independent t-test with Welch’s correction for two sample comparison **p < 0.01, n = 3. (**E**) Immunoblot analysis of LDLR and BMP1 in cell lysates from HepG2 cells treated with siRNA against BMP1 (Ambion) or with a non-targeting control siRNA and then transfected either with an empty vector, BMP1-FLAG or BMP1 E_214_Q-FLAG constructs. Blot images were cropped to highlight the FL and CTF band of LDLR using the AF2148 antibody and BMP1 using the AF1927 antibody. Full blot images are presented in the Supplementary western blot dataset. (**F**) Densitometric analysis of FL LDLR and the CTF from (**E**). Data expressed as percentage of NT siRNA control (not shown in graph) and shown as mean ± SEM, statistical analysis performed using an ANOVA with Bonferoni post-hoc pairwise analysis **p < 0.01, ***p < 0.001, n = 3.

In order to confirm a role for BMP1 in the proteolytic processing of LDLR, siRNA knockdown, using a Smartpool of 4 individual siRNA target sequences, of endogenous BMP1 was performed. In HepG2 cells both the 160 kDa LDLR and the 120 kDa CTF are readily detectable in the cell lysate and the 36–40 kDa NTF is detectable in the conditioned media (Fig. 3A). Treatment of the cells with siRNA targeting BMP1 almost completely abolished the production of both the 120 kDa CTF and the 36–40 kDa NTF (Fig. 3A,B) as compared to the non-targeting siRNA transfected cells. This experiment was repeated using an independent, single siRNA target sequence (Fig. 3C,D). Addition of BMP1 siRNA significantly decreased the relative mRNA expression by 68.7% relative to a non-targeting siRNA sequence, and this knockdown of BMP1 almost completely abolished the production of both the 120 kDa CTF and the 36–40 kDa NTF (Fig. 3C). To further demonstrate that the cleavage of LDLR is a result of BMP1 activity, we performed a rescue experiment using a BMP1-FLAG construct transfected into HepG2 cells following siRNA knockdown and compared this to the transfection of an inactive mutant of BMP1 where the glutamate in the active site motif (His-Glu-Xaa-Xaa-His) was mutated to glutamine (E_214_Q), which renders the enzyme catalytically inactive^15^. Expression of wild type BMP1, following BMP1 siRNA knockdown, resulted in a significant decrease in the full length LDLR with a corresponding significant increase in the 120 kDa CTF, indicating BMP1mediated cleavage of LDLR (Fig. 3E,F). In contrast, expression of the catalytically inactive mutant of BMP1 (E_214_Q), following BMP1 siRNA knockdown, prevented cleavage of the full length LDLR (Fig. 3E,F).

**Figure 4:**
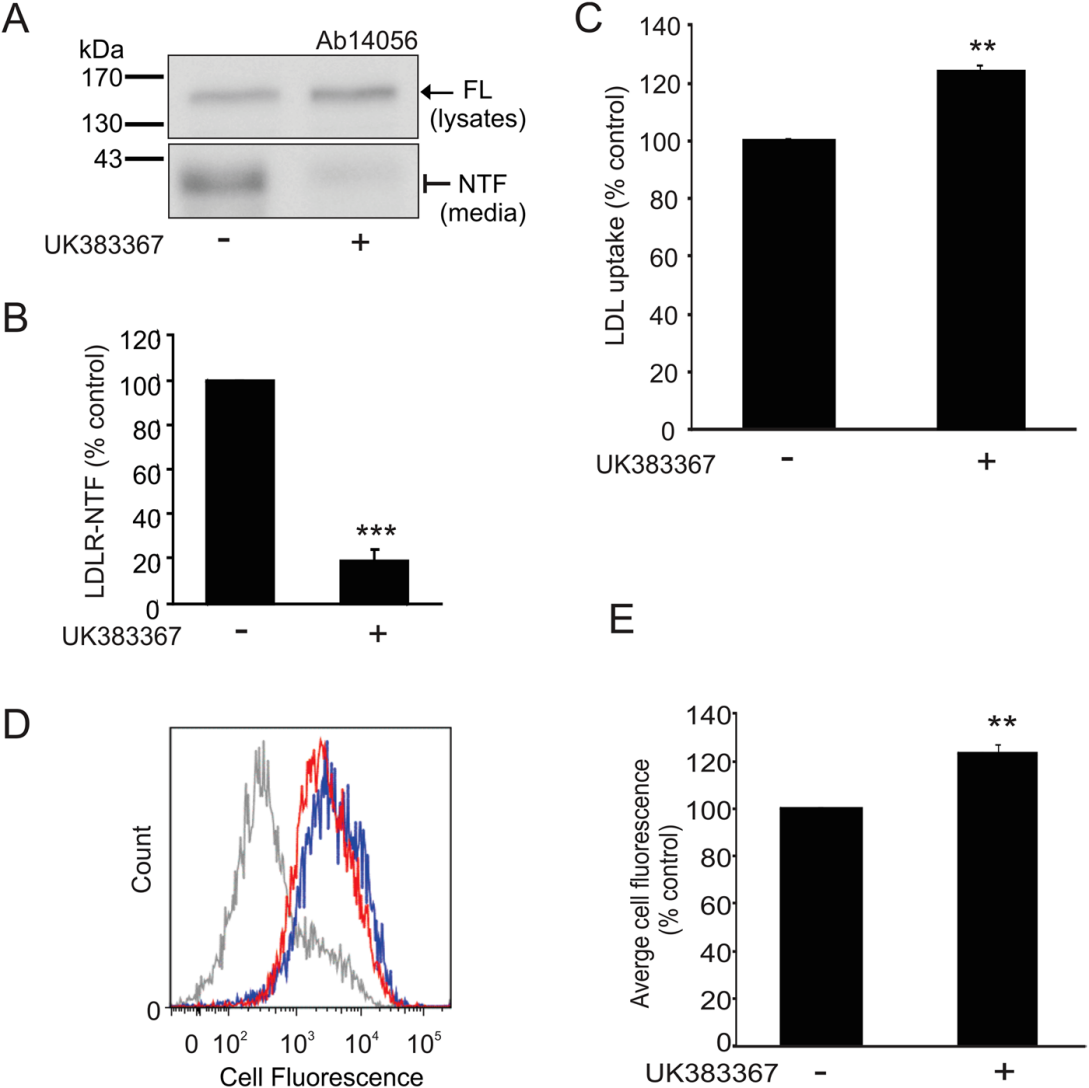
Pharmacological inhibition of BMP1 decreases the proteolytic cleavage of LDLR and increases the cellular uptake of LDL. (**A**) Immunoblot analysis of LDLR in cell lysates and conditioned media from HepG2 cells treated with inhibitor UK383367 (10 μM) for 18 h. Blot images were cropped to highlight the FL and NTF bands in cell lysates and conditioned media, respectively, using the Ab14056 antibody. Full blot images are presented in the Supplementary western blot dataset. (**B**) Densitometric analysis of the NTF from (**A**). Data shown as mean ± SEM, ***p < 0.001, n = 3. (**C**) BODIPY^®^-LDL uptake in HepG2 cells treated with or without inhibitor UK383367 (10 μM) for 6 h. Data expressed as percentage of control and shown as mean ± SEM, statistical analysis performed using a Student’s T-test **p < 0.01, n = 3. (**D**) Representative data from flow cytometry showing the change in cell surface fluorescence of LDLR between HepG2 cells treated with inhibitor UK383367 (10 μM) for 18 h (blue line) and untreated cells (red line); primary antibody (Ab14056) only (grey line). (**E**) Quantitation of the cell surface expression of LDLR in HepG2 cells treated with or without UK383367 as analysed by flow cytometry in (**D**). Data expressed as percentage of control and shown as mean ± SEM, statistical analysis performed using a Student’s T-test **p < 0.01, n = 3.

In addition to genetic manipulation of BMP1 expression, we also used pharmacological inhibition of BMP1 in HepG2 cells. Treatment of the cells with UK383367 to inhibit BMP1 significantly decreased the amount of the 36–40 kDa NTF (Fig. 4A,B) as compared to the untreated cells. The BMP1 inhibitor significantly increased LDL uptake in the cells (Fig. 4C). Although inhibition of BMP1 nearly completely blocked the cleavage of LDLR (Fig. 4B), LDL uptake was only increased by 25% (Fig. 4C). Cell-surface LDLR levels were determined using flow cytometry to ascertain whether this was due to only a proportion of the total amount of full-length LDLR being at the cell surface. Following BMP1 inhibition, cell surface LDLR levels were increased by only 23% (Fig. 4D,E), indicating that the increase in LDL uptake correlates with the increase in cell-surface full-length LDLR.

### The catalytic activity of BMP1 is required for the proteolytic cleavage of LDLR and modulation of cellular LDL uptake

**Figure 5:**
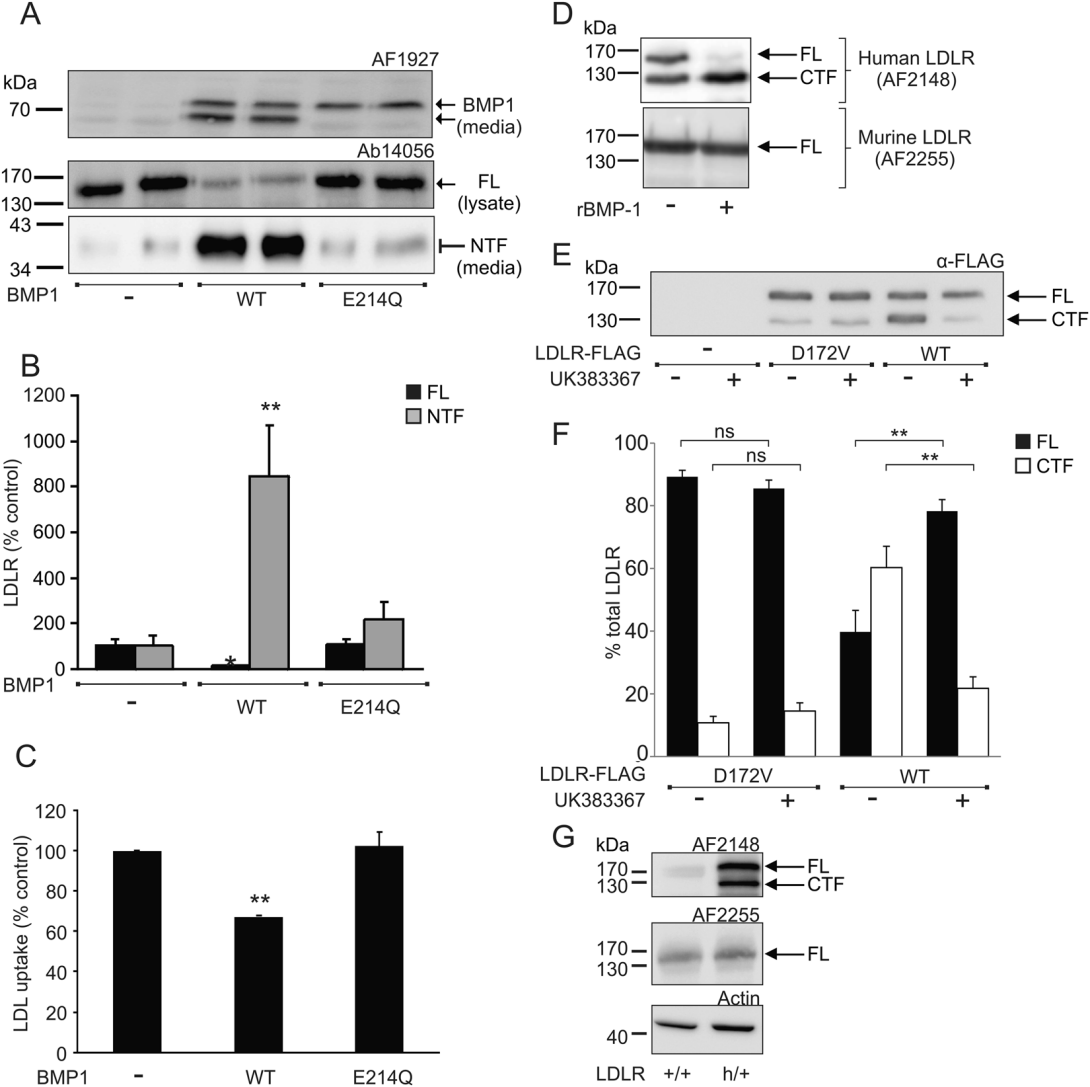
Cleavage of LDLR requires the catalytic activity of BMP1 and human but not murine LDLR is proteolytically cleaved by BMP1 *in vivo*. (**A**) Immunoblot analysis of BMP1 in conditioned media and of LDLR in cell lysates and conditioned media from SW13 cells expressing wild-type (WT) or mutated (E_214_Q) BMP1. Blot images were cropped to highlight BMP-1 bands in conditioned media and the FL and NTF bands in cell lysates and conditioned media, respectively, using the Ab14056 antibody. Full blot images are presented in the Supplementary western blot dataset. (**B**) Densitometric analysis of FL and the NTF from (**A**). Data expressed as percentage of control and shown as mean ± SEM, statistical analysis performed using an ANOVA with Bonferoni post-hoc pairwise analysis *p < 0.05, **p < 0.01, n = 4. (**C**) BODIPY^®^-LDL uptake in SW13 cells expressing either WT or E_214_Q BMP1. Data expressed as percentage of control and shown as mean ± SEM, statistical analysis performed using an ANOVA with Bonferoni post-hoc pairwise analysis **p < 0.01, n = 3. (**D**) Immunoblot analysis of rhLDLR (500 ng) (top panel) and recombinant mouse LDLR (500 ng) (bottom panel) following incubation with rBMP1 (100 ng) for 1 h. Blot images were cropped to highlight the FL and CTF bands in human LDLR using the AF2148 antibody and the FL band only in mouse LDLR using AF2255 antibody. Full blots are not available for this figure. (**E**) Immunoblot analysis of LDLR in lysates from HepG2 cells expressing either wild-type (WT) or mutated (D_172_V) LDLR and incubated with or without inhibitor UK383367 (10 μM) for 18 h. The blot image was cropped to highlight the FL and CTF bands using the anti-FLAG antibody. Full blot images are presented in the Supplementary western blot dataset. (**F**) Densitometric analysis of FL -LDLR and the 120 kDa CTF from (**E**). Data expressed as percentage of control and shown as mean ± SEM, statistical analysis performed using an ANOVA with Bonferoni post-hoc pairwise analysis **p < 0.01, n = 3. (**G**) Immunoblot analysis of human LDLR (top panel) and mouse LDLR (bottom panel) in liver lysates from either wild-type LDLR^+/+^ or transgenic LDLR^h/+^ mice. Blot images were cropped to highlight the FL and CTF bands using the AF2148 antibody and the FL band only in mouse LDLR using AF2255 antibody. Full blot images are presented in the Supplementary western blot dataset.

In the human adrenal SW13 cell line LDLR appears as a single protein of 160 kDa suggesting that BMP1 is either absent or expressed at a very low level in these cells (Fig. 5A). Following transfection of the SW13 cells with the cDNA encoding BMP1, there was a significant reduction in the 160 kDa full length LDLR and a concomitant increase in the 36–40 kDa NTF in the conditioned medium (Fig. 5A,B). Expression of BMP1 in these cells significantly reduced the uptake of fluorescently-labelled LDL particles (by 33%, p < 0.01) (Fig. 5C). Expression of the catalytically inactive E_214_Q BMP1 mutant failed to cleave LDLR (Fig. 5A,B) and did not reduce LDL uptake in the cells (Fig. 5C). Together with the action of the site-directed inhibitor (as shown in Fig. 5), these data indicate that the catalytic activity of BMP1 is required for its proteolytic cleavage of LDLR and that BMP1 mediated cleavage of LDLR modulates cellular LDL uptake.

### Human but not murine LDLR is proteolytically cleaved by BMP1 *in vivo*

Comparison of the protein sequences of human, mouse and rat LDLR indicate that Asp_172_ in the P_1_′ position of the BMP1 cleavage site in the human receptor is replaced with Val in mouse LDLR. As this Asp residue appears to be critical for recognition and cleavage of substrates by BMP1^16^, it raised the possibility that rodent LDLR is not cleaved by BMP1. In support of this, commercially available murine LDLR (R&D Systems) migrates as a single polypeptide of 160 kDa on SDS-PAGE (Fig. 5D), in contrast to the two polypeptides observed for human LDLR (even though both receptors are produced in murine myeloma cells). The recombinant murine and human LDLRs were incubated with recombinant BMP1 and, as previously noted, BMP1 cleaved the human LDLR to generate the 120 kDa CTF (Fig. 5D). However, the murine receptor was not cleaved even after prolonged incubation with BMP1 (Fig. 5D). To confirm that the Asp was critical for cleavage of LDLR by BMP1, this residue was mutated to Val in human LDLR (D_172_V). When expressed in HepG2 cells, wild-type human LDLR was cleaved in a BMP1-dependent manner, however, BMP1 failed to cleave the mutated D_172_V receptor (Fig. 5E,F). In liver lysates from transgenic mice in which one allele of the murine LDLR is replaced with human LDLR (LDLR^h/+^ transgenic mice)^17^ both the 160 kDa full-length and the 120 kDa CTF forms of human LDLR were observed, while only the 160 kDa murine LDLR was observed in the liver lysates of wild type (LDLR^+/+^) mice (Fig. 5G), confirming that human LDLR is proteolytically cleaved in its ectodomain *in vivo* and that murine LDLR is resistant to such cleavage. Together these data confirm that human LDLR is proteolytically cleaved *in vivo* whereas murine LDLR is not cleaved by BMP1. The lack of cleavage of murine LDLR is likely due to the substitution of Val for Asp at the BMP1 cleavage site.

## Discussion

Here we report the proteolytic cleavage of LDLR by BMP1 as a novel mechanism for post-translational regulation of LDLR function and cellular LDL cholesterol levels. Initially we identified that human LDLR is proteolytically cleaved between Gly_171_ and Asp_172_ (…VFQG_171_↓DSSP…) within the linker region between LA repeats 4 and 5 in its extracellular ligand binding domain. This cleavage results in the production of a 120 kDa membrane bound CTF, previously identified in HepG2 cells but considered to be the immature, unglycosylated form of the receptor^8,18^. Deglycosylation indicated that the 120 kDa CTF is not an immature, unglycosylated form of the full-length LDLR and N-terminal sequencing revealed that it lacks LA repeats 1–4. The 120 kDa CTF likely corresponds to a minor band of similar molecular weight observed on SDS PAGE, designated “band X” that was not recognised by an antibody directed towards a region near the N-terminus of the receptor and was thus suggested to be a proteolytically processed form of the receptor that had lost the N-terminal LDL binding domain^19^.

During crystallization of LDLR with PCSK9, adventitious proteolysis of the LDLR at the Gly_171_↓Asp_172_ peptide bond was observed upon expression of the receptor in HEK293 cells^11^. This “receptor degradation” was prevented when purification and crystallization were carried out in the presence of undefined protease inhibitors^11^. Another study also reported the proteolytic cleavage of LDLR to produce a CTF lacking LA repeats 1–4, although the precise cleavage site or identify of the protease responsible were not determined^12^. In addition to the 120 kDa CTF, we also identified a 36–40 kDa NTF fragment of LDLR. Although the molecular mass of LA repeats 1–4 is predicted to be 19 kDa, a similar fragment produced by cleavage of the LDLR with α-chymotrypsin resolved with a higher apparent molecular mass of 36 kDa on SDS-PAGE^20^.

BMP1 is a secreted zinc-dependent metalloproteinase belonging to the group of matrix metalloproteinases of the astacin family^21^. The structure of BMP1 is highly conserved across species and the protease is required for the maturation of fibrillar collagen from procollagen by proteolytic removal of the C-terminal propeptides^21^. BMP1 is also involved in the formation of the extracellular matrix through the activation of numerous structural proteins and enzymes^21^, and in the generation of growth factors involved in morphogenesis, including the cleavage and activation of members of the TGF-β family^22^. We established that the catalytic activity of BMP1 is required for LDLR cleavage through use of an active site directed inhibitor and through mutation of a critical residue in the active site of the protease. This, together with the observation that recombinant BMP1 can directly cleave recombinant LDLR, indicates that LDLR is a substrate for BMP1, rather than BMP1 acting on another protein that regulates LDLR proteolysis or acting as a chaperone to traffic LDLR to another cellular compartment (e.g. lysosomes) where cleavage by other proteases occurs.

The action of BMP1 on LDLR significantly compromised the ability of the receptor to bind and endocytose LDL. Binding of BODIPY-LDL to the 120 kDa CTF was significantly reduced compared to binding to the full-length LDLR and expression of the 120 kDa CTF construct in CHO-A7 cells lacking endogenous LDLR demonstrated that, although present at the cell surface, the CTF displayed reduced binding and uptake of LDL into cells compared to full-length LDLR. This is in agreement with previous work demonstrating that LDLR lacking LA repeats 1–4 is unable to bind and endocytose LDL^12,23,24^. Truncated LDLR has been reported to bind a different class of lipoprotein, β-very low-density lipoprotein (β-VLDL)^23,24^, suggesting that cleavage of the LDLR by BMP1 may act to remodel the function of the receptor to enhance uptake of other classes of lipoprotein.

Inhibition of BMP1 cleavage of LDLR by the molecular chaperone RAP, which associates with LDLR in the endoplasmic reticulum to ensure proper folding and trafficking through the secretory pathway^25^, indicates that proteolysis must occur following dissociation of RAP in the medial-Golgi. That LDLR bound to LDL is also resistant to cleavage by BMP1 implies that BMP1 likely cleaves LDLR later in the secretory pathway or at the cell surface before binding and internalising LDL. The presence of the 120 kDa CTF at the cell surface, as evidenced by its ability to be surface biotinylated, is consistent with this assumption. Although pharmacological inhibition or genetic knockdown of BMP1 almost totally inhibited LDLR cleavage, LDLR cell surface expression and LDL uptake were only increased by ~20% suggesting tight regulation of the amount of receptor expressed at the cell surface as observed in previous studies^26,27^.

Given the high level of sequence homology between human and murine LDLR, it was unexpected to observe that murine LDLR is resistant to BMP1 cleavage. Sequence alignment revealed that the critical Asp required in the P1′ position of the BMP1 cleavage site in its substrates is Val in murine LDLR and mutation of the Asp to Val in human LDLR rendered the protein completely resistant to cleavage by BMP1. Consistent with this experimental observation, 35 out of 41 of the known substrate cleavage sites of BMP1 incorporate Asp in the P_1_′ site and none have Val at this position^16,21^ (https://www.ebi.ac.uk/merops). The resistance of murine LDLR to cleavage by BMP1 was confirmed by the presence of a single band of LDLR in western blots of mouse liver samples. In contrast, in a transgenic mouse model in which one of the mouse LDLR alleles has been replaced with a human LDLR allele^17^, human LDLR appeared as the 160 kDa full-length receptor and the 120 kDa CTF. Together with the observation of a 120 kDa CTF in human liver samples, this demonstrates that human LDLR is proteolytically cleaved *in vivo*, likely, by BMP1.

As BMP1 cleaves a number of extracellular substrates including structural proproteins, proenzymes and latent growth factors, using inhibitors targeted to the active site of the protease to increase LDLR levels and hence cellular LDL uptake could have undesirable side effects. BMP1 has previously been implicated in lipid metabolism as the protease responsible for cleaving human proapolipoprotein A1, the protein component of high-density lipoprotein particles^28^. BMP1 cleaves newly secreted proapolipoprotein A1 to its phospholipid-binding form, promoting the formation of functional HDL^28,29^. Thus inhibition of BMP1 might have LDL lowering effects but also HDL lowering effects. The activity of BMP1 on procollagen and pro-apolipoprotein A1 is specifically increased several-fold by procollagen C-proteinase enhancer-2 (PCPE2) that forms a ternary complex with the protease and its substrate^29,30^. Whether PCPE2 interacts with LDLR and facilitates its cleavage by BMP1 is to be determined. Secondary binding sites (exosites) are sites outside of the active site that participate in substrate recognition and binding by an enzyme. Although full length LDLR is efficiently cleaved by recombinant BMP1, a 10-mer peptide (YVFQGDSSPC) based on the cleavage site in human LDLR was not cleaved (data not shown), suggesting that sites elsewhere on LDLR and/or BMP1 are required for substrate recognition and binding^31^. This raises the distinct possibility that BMP1 and LDLR interact via an exosite and that this association may be enhanced by other proteins such that disruption of this interaction could be a targeted therapeutic approach to selectively block BMP1 action on LDLR.

A limitation of our study is whether BMP1 may prevent cellular cholesterol overload and contribute to the regulation of LDLR at the cell surface *in vivo*. In an attempt to modulate BMP1 levels *in vivo*, the LDLR^h/+^ transgenic mice were crossed with mice heterozygous for BMP1 (BMP1^+/−^) (as the homozygous BMP1 knockout is embryonic lethal)^32^, to generate LDLR^h/+^ BMP1^+/−^ and LDLR^h/+^ BMP1^+/+^ transgenic lines. However, in the BMP1 heterozygous mice, BMP1 protein levels were unchanged as compared to the wild type mice (Supplementary Fig. 2). Pharmacological inhibition of BMP1 in the LDLR^h/+^ transgenic mice using the inhibitor, UK383367, was not feasible due to the short half-life (49 min in plasma) and relative insolubility of this compound in aqueous solution^33^.

Finally, we have described a new cellular mechanism that controls the amount of LDLR at the cell surface and therefore LDL uptake. Further work is needed to assess the impact of the BMP1 cleavage of LDLR on plasma LDL cholesterol *in vivo*. However, it is interesting to note that integrative genomic, epigenomic and transcriptomic profiling of perturbed coronary artery smooth muscle cells and tissues identified BMP1 as one of the top seven candidate loci contributing to coronary artery disease^34^. Thus BMP1 may play a critical role in atherosclerotic cardiovascular disease through an as yet unknown mechanism within vascular cells, as well as through modulating cholesterol metabolism in hepatocytes.

## Methods

### Human liver preparation

Human liver samples were obtained from the Manchester Foundation Trust Biobank, Manchester University NHS Foundation Trust, UK (REC approval number 14/NW/1260) and were snap frozen and stored at −80 °C. Samples were homogenised using an electric homogeniser in RIPA buffer (150 mM NaCl, 1% (v/v) Nonidet P-40, 0.5% (w/v) sodium deoxycholate, 50 mM Tris pH 8.0) containing protease inhibitor cocktail (Roche). Homogenates were centrifuged at 13,000 g for 10 min at 4 °C, the supernatant was removed and centrifuged again at 10,000 g for 30 min at 4 °C. The supernatant was then taken as the clarified lysate and either used immediately or stored at −80 °C before further analysis. The protein concentration of the samples was determined using bicinchoninic acid (Sigma Aldrich) and 100 µg of each sample was used for SDS-PAGE and western blotting. Clarified lysates (100 µg) from human liver or from HepG2 cells were deglycosylated using the Protein Deglycosylation Mix II, Cat #P6044 (New England Biolabs, Hitchin, Hertfordshire, UK), according to the manufacturer’s instructions. This deglycosylation protocol removes all *N*-linked and simple *O*-linked glycans as well as some complex *O-*linked glycans.

### Animals

Human LDLR transgenic (LDLR^h/+^) mice have been described previously^17^. Mice heterozygous for BMP-1 (BMP-1^+/−^), as described previously^32^ were crossed with the LDLR^h/+^ mice to generate LDLR^h/+^ BMP-1^+/+^ and LDLR^h/+^ BMP-1^+/−^ mice. All mice were housed in standard conditions (temperature of 20 ± 2 °C; humidity, 55 ± 5%; 12 h light/12 h dark cycle) and were given *ad libitum* access to a standard laboratory diet (RM1, Special Diet Services, UK) and water. All experimental procedures using animals were conducted in accordance with the United Kingdom Animals (Scientific Procedures) Act, 1986 and approved by the Home Office and the local Animal Ethical Review Group, University of Manchester. At 8 weeks of age, mice were maintained on a high cholesterol (1.25%) containing diet (code 820190, Special Diet Services, Witham, Essex, England) for 2 weeks. Mice were then fasted for 6 h during the light phase and sacrificed using CO_2_ asphyxiation followed by removal of their livers. Mouse livers were stored at −80 °C until further analyses.

### Mouse liver preparation

Mouse livers were homogenised (150 mg/ml wet weight) using a mechanical homogeniser in RIPA buffer containing protease inhibitor cocktail. Samples were centrifuged at 10,000 g for 10 min at 4 °C and the supernatant removed and centrifuged again at 10,000 g for 30 min at 4 °C to recover the lysate. The clarified lysates were stored at −80 °C. Protein concentration of the lysate samples were determined using bicinchoninic acid (Sigma-Aldrich).

### SDS-PAGE and western blotting

Samples were resolved on 7–17% polyacrylamide gels and transferred to Hybond-P polyvinylidene difluoride membrane (GE Healthcare, Little Chalfont, Buckinghamshire, UK). The membranes were then blocked for 1 h in phosphate-buffered saline (PBS) containing 0.1% (v/v) Tween (PBS-T), with 2% (w/v) bovine serum albumin (BSA) and 5% (w/v) dried milk powder at room temperature. Membranes were incubated overnight at 4 °C in primary antibody diluted in 2% BSA in PBS-T: goat anti-human LDLR (AF2148), goat anti-human BMP1 (AF1927), goat anti-mouse LDLR (AF2255) (R&D systems), chicken anti-LDLR (Ab14056) (Abcam, Cambridge, UK) at a dilution of 1:1000, anti-FLAG M2 antibody (Sigma-Aldrich) at a dilution of 1:2500 and anti-actin antibody (Sigma-Aldrich) at a dilution of 1:5000. Membranes were washed in PBS-T and then incubated with the appropriate peroxidase-conjugated secondary antibody, rabbit anti-goat, rabbit anti-chicken, donkey anti-chicken or rabbit anti-mouse (Sigma-Aldrich) at a 1:4000 dilution in 2% BSA in PBS-T. Membranes were washed in PBS-T with a final wash in PBS before detection by the enhanced chemiluminescence detection method using Pierce ECL Western Blotting Substrate (Fisher Thermo Scientific, Cramlington, Northumberland, UK). Blots were developed using a Fujifilm LAS3000 Imager or a Syngene Gbox XT4 with multiple exposures used to obtain optimised images for each of the protein bands of interest. Alterations in contrast were made across the whole blot before the analysis of blot densitometry using Aida 2D densitometry (Elysia Raytest) or Genetools software (Syngene). Blot images were processed using CorelDRAW graphics and images cropped to highlight bands of interest for clarity; full-size blot images are shown in the supplementary western blot data file.

### *In vitro* proteolysis of recombinant LDLR

Recombinant human LDLR (rhLDLR; 500 ng; 100 ng/µl) (R&D systems, Abingdon, UK) was incubated with increasing concentrations (0 to 12.5 ng) of recombinant BMP1 (rBMP1) (R&D Systems) in HEPES buffer (25 mM HEPES, 0.01% Brij 35, pH 7.5) in a total volume of 15 µl for 1 h at 37 °C. For BMP1 inhibitor experiments, 500 ng rhLDLR was incubated with or without 12.5 ng rBMP1 in the absence or presence of 10 µM UK383367 (3-(Aminocarbonyl)-β-(3-cyclohexylpropyl)-*N-*hydroxy-1,2,4,-oxadiazole-5-propanamide) (Tocris Bioscience, Bristol, UK) in a total volume of 15 µl for 1 h at 37 °C. For LDL and receptor-associated protein (RAP) experiments 500 ng rhLDLR was pre-incubated for 30 min on ice in the absence or presence of 5 µg LDL (Invitrogen Life Technologies, Paisley, UK), 7.14pmoles RAP (2:1 RAP:LDLR) (Calbiochem, Merck Chemicals, Nottingham, UK) or 10 µM UK383367 before addition of 12.5 ng rBMP1 and further incubation in a total volume of 15 µl for 1 h at 37 °C. For comparison of human and mouse LDLR cleavage, 500 ng rhLDLR or 500 ng recombinant mouse LDLR (R&D Systems) were incubated with 100 ng rBMP1 in HEPES buffer for 1 h at 37 °C. Reactions were stopped by addition of 2x dissociation buffer (140 mM Tris-HCl, 10% (w/v) sodium dodecyl sulphate, 20% (v/v) glycerol, 100 mM dithiothreitol, 0.005% (w/v) bromophenol blue, pH 6.8) and samples were heated at 95 °C for 5 min before SDS-PAGE and western blot analysis.

### Dot blotting

rhLDLR-His (5 µg) was incubated in the presence or absence of rBMP1 (200 ng) at 37 °C for 1 h. Samples (0.5 µg) from the two treatments were spotted onto nitrocellulose membrane along with samples (0.5 µg) of PCSK9-FLAG (GlaxoSmithKline, Stevenage, UK) and BACE1-His (R&D Systems) and allowed to dry for 20 min. Membranes were incubated overnight in blocking buffer (PBS containing 5% (w/v) BSA) and then were incubated with BOPIDY-LDL (1 µg/ml) for 3 h at room temperature in the dark. Membranes were washed in DPBS and bound BODIPY-LDL was detected using a Biorad molecular imager FX (Bio-Rad Laboratories, Hercules, California, USA). Membranes were stripped and re-probed with either a mouse anti-His or a mouse anti-FLAG HRP-conjugated primary antibody for the ECL detection of His-tagged and FLAG-tagged proteins, respectively. Densitometric analysis of dots was performed using Image J software (National Institute of Health).

### Cell culture

Human hepatocellular carcinoma (HepG2) cells were maintained in Advanced Minimum Essential Medium (Advanced-MEM) (Invitrogen Life Technologies) containing 10% (v/v) foetal bovine serum (FBS) (Biosera, Uckfield, UK) and 2mM L-glutamine (Gibco, Paisley, UK). Human adrenal carcinoma (SW13) cells were maintained in Dulbecco’s modified Eagle’s Medium (DMEM) (Lonza, Basel, Switzerland) containing 10% (v/v) FBS. Chinese hamster ovary cells lacking LDLR (CHO-A7) were obtained from Professor N. G. Seidah (University of Montreal, QC, Canada) and maintained in Ham’s F12 medium containing L-Glutamine (Lonza) and supplemented with 10% (v/v) FBS. All cell lines were cultured in a humidified atmosphere of 5% CO_2_ at 37 °C. Cells were passaged using trypsin EDTA (Lonza). HepG2 cells were grown to 60–70% confluency in a 6-well plate and incubated in 2.5 ml of Opti-MEM + GlutaMAX (Gibco) with or without the addition of 10 µM UK383367 for 18 h before cells and the conditioned cell medium were harvested.

### Plasmids, site-directed mutagenesis and transient transfection

cDNA encoding either the full-length human LDLR or its BMP1 truncated 120 kDa CTF with a 5′ signal peptide sequence and a 3′ FLAG tag were inserted into the mammalian expression vector pcDNA3.1(+) within the 5′ Hind III and 3′ Bam HI sites. CHO-A7 cells were transfected with FL and CTF constructs using the *Trans*IT-LT1 transfection reagent (Mirus, Madison, USA) according to the manufacturer’s protocol. Cells were incubated for 30 h before the medium was replaced with serum-free Ham’s F12 medium for a further 18 h before being harvested or fixed.

Full-length human wild-type LDLR cDNA with 3′ FLAG tag sequence in Gateway vector pFastBacMam-2-rfa (GlaxoSmithKline, Stevenage, UK) was used to generate the D_172_V mutant using the Quikchange II Site-Directed Mutagenesis kit (Stratagene, Cambridge, UK). Primers (Sigma-Aldrich, Dorset, UK); forward primer 5′-CGTGTTCCAAGGGGTCAGTAGCCCCTGCTCG-3′ and reverse primer 5′-CGAGCAGGGGCTACTGACCCCTTGGAACACG-3′. HepG2 cells were transfected with WT and mutant LDLR constructs using *Trans*IT-LT1 transfection reagent and cells were incubated for 42 h before being harvested. For BMP1 inhibitor experiments, 10 µM UK383367 was added after 24 h for a further 18 h incubation before harvesting.

Human BMP1 cDNA in pcDNA3.1 (a kind gift from Dr E. Laird, University of Liverpool, UK) was used to generate the E_214_Q mutant using the Quikchange II Site-Directed Mutagenesis kit (Stratagene). Primers (Sigma-Aldrich): forward primer 5′-GGCATTGTGGTCCACCAGCTGGGCCACGTCGTCGGC-3′ and reverse primer 5′-GCCGACGACGTGGCCCAGCTGGTGGACCACAATGCC-3′. SW13 cells were transfected with the WT and mutant BMP1 constructs using *Trans*IT-LT1 transfection reagent. Cells were incubated for 48 h before the medium was replaced with Opti-MEM for a further 6 h incubation before harvesting cells and conditioned medium.

Human BMP1-FLAG cDNA in pcDNA3.1 (a kind gift from Dr E. Laird, University of Liverpool, UK) was used to generate the E_214_Q mutant using the Q5 Site-Directed Mutagenesis kit (NEB). Primers (Sigma-Aldrich): forward primer 5′-TGTGGTCCACCAGCTGGGCCA-3′ and reverse primer 5′-ATGCCGAACTTGTCACAGTTCTTG-3′. BMP1-FLAG constructs were used for siRNA knockdown rescue experiments in HepG2 cells as detailed below.

### Cell lysate and conditioned media preparation

Conditioned media samples were concentrated to 200 µl using 10 kDa cut-off Vivaspin filtration columns (Millipore, Billerica, MA, USA). Cells were washed twice in PBS, harvested and pelleted by centrifugation. Cell pellets were lysed, on ice, in RIPA buffer with protease inhibitor cocktail. Lysates were clarified by centrifugation at 13,000 g for 10 min. The protein concentration of the samples was determined using bicinchoninic acid assay (Sigma-Aldrich).

### Cell-surface biotinylation

CHO-A7 cells were grown in 25 cm^2^ flasks and transiently transfected with the cDNA encoding either full-length LDLR or the 120 kDa CTF as described above. Cells were then washed twice in ice-cold PBS and incubated on ice for 20 min in PBS with or without the addition of 0.5 mg/ml EZ-Link Sulfo-NHS-SS-Biotin (Life Technologies, Paisley, United Kingdom) to label cell surface proteins. Cells were washed twice in ice-cold PBS, harvested and cell lysates prepared. Lysates (500 µg total protein) were incubated with 50 µl of streptavidin-agarose beads (Sigma-Aldrich, Dorset, United Kingdom) for 3 h at 4 °C on a rotator to precipitate the biotin-labelled surface proteins. Beads were pelleted by centrifugation, washed three times in ice-cold RIPA buffer and resuspended in 40 µl of dissociation buffer. Samples were heated at 95 °C for 5 min followed by SDS-PAGE and western blot analysis.

### Immunofluorescence microscopy

CHO-A7 cells were seeded at 10,000 cells per well onto coverslips in 24 well plates and transiently transfected with the cDNA encoding either full-length LDLR or the 120 kDa CTF as described above. Cells were then incubated in serum-free medium containing human LDL labelled with 1,1′-dioctadecyl-3,3,3′,3′-tetramethyl-indocarbocyanine perchlorate (Dil-LDL; Life Technologies) (10 µg/ml) for 30 s on ice and fixed using 4% paraformaldehyde for 10 min at room temperature. Cells were blocked in 5% fish skin gelatin (FSG, Sigma-Aldrich) for 1 h at room temperature and then incubated in LDLR antibody, AF2148 diluted 1:100 in 5% FSG. Cells were then washed three times in PBS before incubation with secondary antibody, anti-goat Alexa 488 conjugated IgG diluted 1:1000 in 5% FSG, for 1 h at room temperature. Finally, cells were washed twice in PBS containing 0.2% Tween-20, followed by a PBS only wash and the coverslips were then mounted on glass slides using ProLong Gold Antifade Mountant containing DAPI. Images were acquired on an Olympus IX83 inverted microscope using UV (DAPI), blue (AF2148) and green (Dil-LDL) Lumencor LED excitation, a 60x/1.42 PlanApo N (Oil) objective and the Sedat QUAD (DAPI/FITC/TRITC/Cy5) filter set (Chroma [89000]). The images were collected using a Retiga R6 [Q-Imaging] CCD camera with a Z optical spacing of 0.2 μm. Raw images were then deconvolved using the Huygens Pro software (SVI) and maximum intensity projections of these deconvolved images are shown in the results. Seven images from three independent experiments for each condition were analysed for fluorescence intensity measurements.

### BODIPY-LDL uptake assay

HepG2, SW13 or transfected CHO-A7 cells were seeded onto clear bottomed, black walled 96-well plates in cell culture medium for 24 h before a further 18 h incubation in serum-free culture media. Medium was then replaced with serum-free culture medium containing 10 µg/ml BODIPY [boron dipyrromethene (4,4-difluoro-4-bora-3a,4a-diaza-s-indacene)]-LDL and cells were incubated for 6 h. For BMP1 inhibition, medium contained 10 µM UK383367. To determine cellular BODIPY-LDL uptake the media was removed, the cells washed twice in PBS with a final volume of 50 µl PBS added before measuring the fluorescence in a Synergy HT plate reader using excitation/emission wavelengths of 485 nm/535 nm.

### siRNA knockdown

ON-TARGETplus SMART pool siRNA against human BMP1 (Dharmacon, Thermo Fisher Scientific Biosciences, Northumberland, UK) and a non-targeting sequence (Dharmacon) were used in HepG2 cells. siRNA (50 nM) was delivered as a complex with DharmaFECT-1 transfection reagent (Dharmacon) according to the manufacturer’s protocol. Cells were incubated for 72 h for siRNA knockdown, washed and then further incubated for 24 h in Opti-MEM before harvesting cells and conditioned media. An additional, single siRNA sequence for human BMP1 was obtained for verification experiments (Ambion). This siRNA was used at a concentration of 25 nM and delivered as above. Cells were incubated for 24 h for siRNA knockdown, washed and then further incubated for 24 h in Opti-MEM before harvesting conditioned media for western blotting and cells for western blotting and qPCR analysis. For rescue experiments, 25 nM siRNA against BMP1 (Ambion) was delivered, as above, to HepG2 cells with 24 h initial incubation. WT and mutant BMP1-FLAG constructs were then delivered using *Trans*IT-LT1 transfection reagent and incubated for a further 48 h. Cells were then harvested for western blot analysis.

### qPCR

Following BMP1 knockdown by siRNA (25 nM, 48 h, Ambion), HepG2 cells were harvested and RNA was extracted using the RNeasy Plus Kit (QIAgen) according to the manufacturer’s instructions. cDNA was synthesised according to the manufacturer’s instructions, using 1 µg of prepared RNA using the iScript cDNA Synthesis Kit (BioRad). BMP1 mRNA expression levels were then quantified using real-time qPCR using the SYBR green method (Applied Biosystems) with the following primer sequences: forward primer 5′-ACTACATGGAGCTCTTCGACG-3′ and reverse primer 5′-TCATCCGAGTGGAACTTCACC-3′. Samples were run, in triplicate, using the Quantstudio 3 (Life Technologies) and relative expression was calculated using ribosomal mRNA expression as baseline control.

### Flow cytometry

HepG2 cells were grown to ~80% confluency and then incubated either with or without 10 µM UK383367 in Advanced MEM for 18 h. Cells were harvested using PBS (without calcium and magnesium ions) containing 2.5 mM EDTA, counted and resuspended at a cell density of 5 × 10^6^ cells/ml in PBS, 2.5 mM EDTA. Cells were blocked in blocking buffer (PBS, 2.5 mM EDTA, 2% (v/v) FSG) for 30 min at 4 °C before incubation with primary antibody, Ab14056 (1:1000 dilution in blocking buffer), for 1 h at 4 °C. Cells were recovered by centrifugation and washed in PBS, 2.5 mM EDTA before incubation with fluorescent conjugated Alexa Fluor 647 anti-chicken secondary antibody (Invitrogen Molecular Probes, Paisley, UK) (1:1000 dilution in blocking buffer). Cells were recovered by centrifugation and washed in PBS, 2.5 mM EDTA before final resuspension in 0.5 ml cold PBS, 2.5 mM EDTA. Prior to fluorescence measurement, 1 µl of DAPI was added and the fluorescence measured using a BD-LSR Fortessa flow cytometer. Ten thousand events for each condition were measured, having gated the cell population to ensure that only live cells were monitored.

### Statistical analysis

Data are shown as mean ± standard error of the mean (SEM). Independent *t*-tests with Levene’s test for equal variance were performed for data comparison between two sets of data and one-way ANOVA with either a Tukey or Bonferroni post-hoc correction for pairwise comparison of multiple data sets. Comparison of mRNA relative expression levels from qPCR analysis was performed using a Welch’s t-test. p < 0.05 was considered significant (*p < 0.05; **p < 0.01, ***p < 0.001). The data were analysed using the Statistical Package for Social Sciences (SPSS) version 20 and 22, (IBM, Chicago, IL, USA) and GraphPad Prism version 7.0, (GraphPad Software Inc. California, USA).

## Supplementary information

Supplementary figures

## Data Availability

The datasets generated and analysed during the current study are available from the corresponding author on reasonable request.
