# Author Correction: Proteolysis of the low density lipoprotein receptor by bone morphogenetic protein-1 regulates cellular cholesterol uptake

**DOI:** 10.1038/s41598-020-59765-y

**Published:** 2020-02-11

**Authors:** Sreemoti Banerjee, Robert J. Andrew, Christopher J. Duff, Kate Fisher, Carolyn D. Jackson, Catherine B. Lawrence, Nobuyo Maeda, Daniel S. Greenspan, Katherine A. B. Kellett, Nigel M. Hooper

**Affiliations:** 1 School of Biological Sciences, Faculty of Biology, Medicine and Health, University of Manchester, Manchester Academic Health Sciences, Manchester, M13 9PT UK; 2 School of Molecular and Cellular Biology, Faculty of Biological Sciences, University of Leeds, Leeds, LS2 9JT UK; 3 Department of Pathology and Laboratory Medicine, University of North Carolina, Chapel Hill, North Carolina USA; 4 Department of Cell and Regenerative Biology, School of Medicine and Public Health, University of Wisconsin, Madison, WI USA; 5 Present Address: Jack Birch Unit for Molecular Carcinogenesis, Department of Biology, University of York, York, YO10 5DD UK; 6 Present Address: Department of Neurobiology, The University of Chicago, Chicago, IL 60637 USA; 7 Present Address: Department of Clinical Biochemistry, University Hospitals of North Midlands NHS Trust, Stoke-on-Trent, ST4 6QG UK

Correction to: *Scientific Reports* 10.1038/s41598-019-47814-0, published online 06 August 2019

This Article contains an error in Figure 2A, where there was an image processing error.

The product datasheet for antibody AF2148 has been updated, and the target region of this antibody has been specified as Asp193-Arg788 (https://www.rndsystems.com/products/human-ldlr-antibody_af2148#product-details). Therefore, the text in the Results, under the subheading “Human LDLR is proteolytically cleaved in its extracellular ligand binding domain”,

“… AF2148 antibody raised against the entire ectodomain of LDLR…”

should read:

“… AF2148 antibody raised against amino acids 193-788 of LDLR…”

Additionally, the text in Figure 1’s legend,

“… antibody AF2148 (R&D Systems) raised against the entire ectodomain of LDLR…”

should read:

“… antibody AF2148 (R&D Systems) raised against amino acids 193-788 of LDLR…”

The correct Figure 2A appears below as Figure 1.

**Figure 1:**
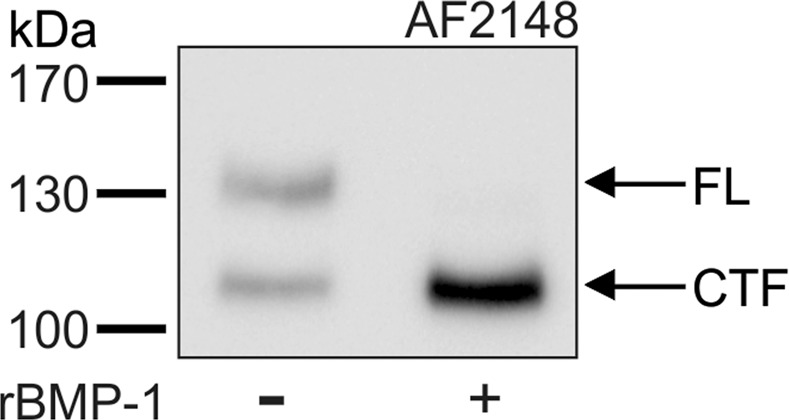
.

