# Supplementary material for: Proteolysis of the low density lipoprotein receptor by bone morphogenetic protein-1 regulates cellular cholesterol uptake
Source: Sci Rep. 2019 Aug 6;9:11416. doi: 10.1038/s41598-019-47814-0 (PMC6684651; doi:10.1038/s41598-019-47814-0)

Proteolysis of the low density lipoprotein receptor by bone morphogenetic protein-1 regulates cellular cholesterol uptake

Sreemoti Banerjee, Robert J. Andrew, Christopher J. Duff, Kate Fisher, Carolyn D. Jackson, Catherine B. Lawrence, Nobuyo Maeda, Daniel S. Greenspan, Katherine A.B. Kellett & Nigel M. Hooper

Figure 1B

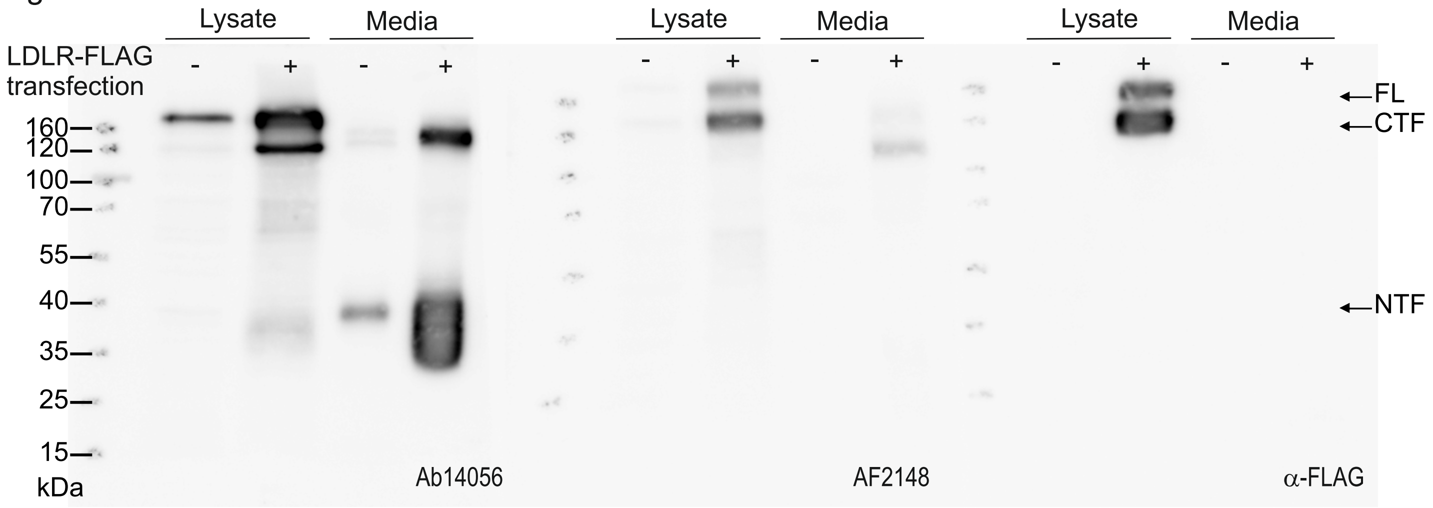

Figure 1D

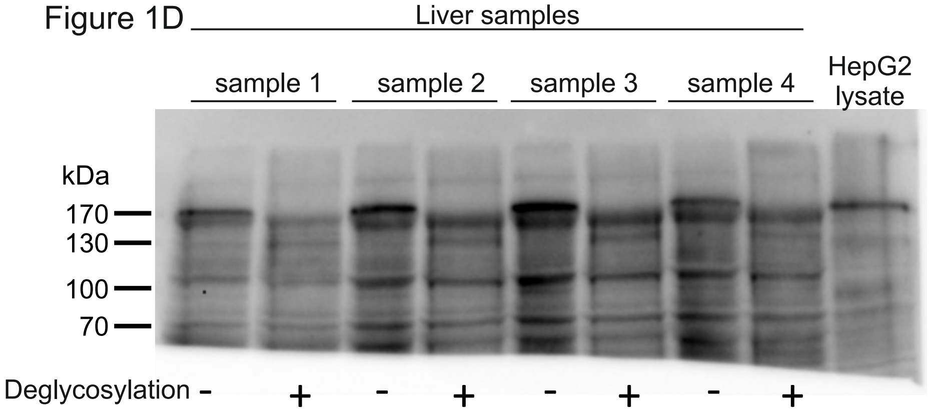

Figure 1E

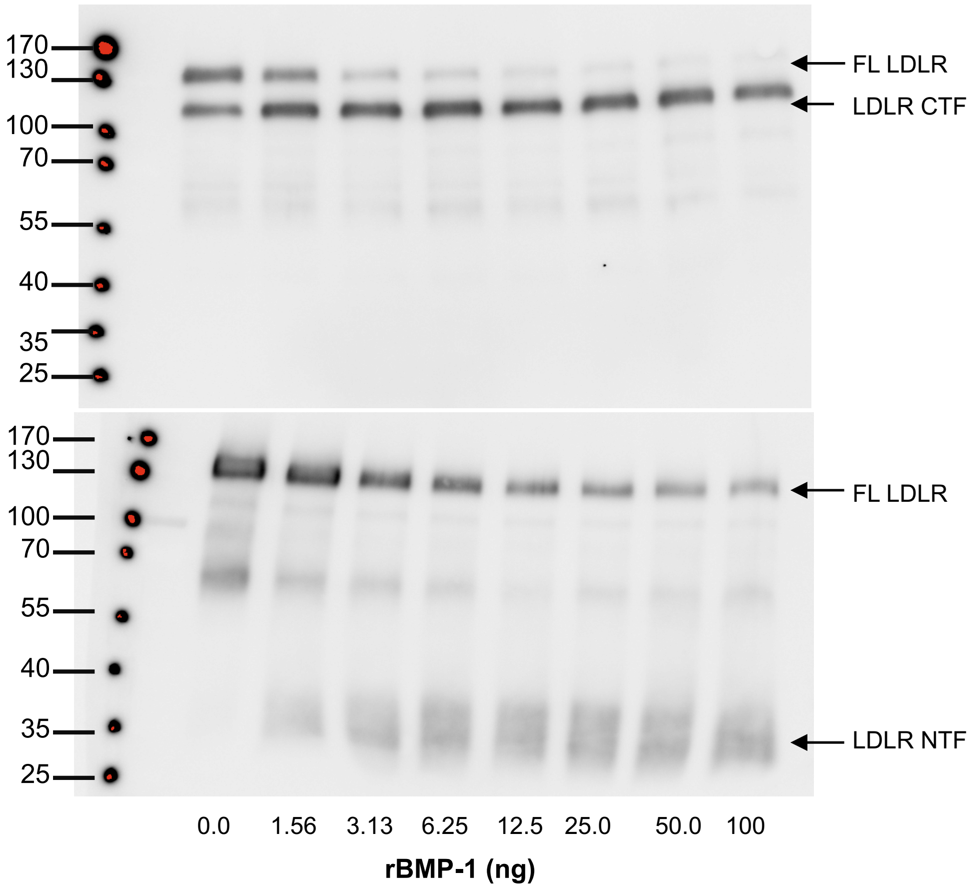

Figure 1F

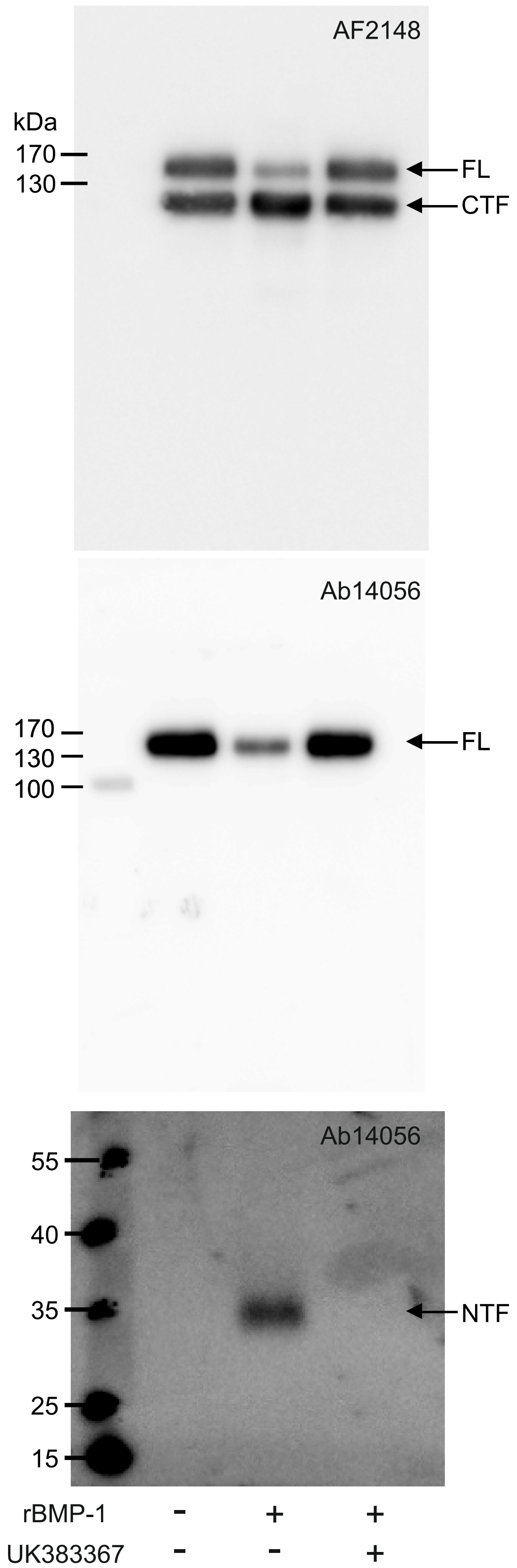

Figure 1G

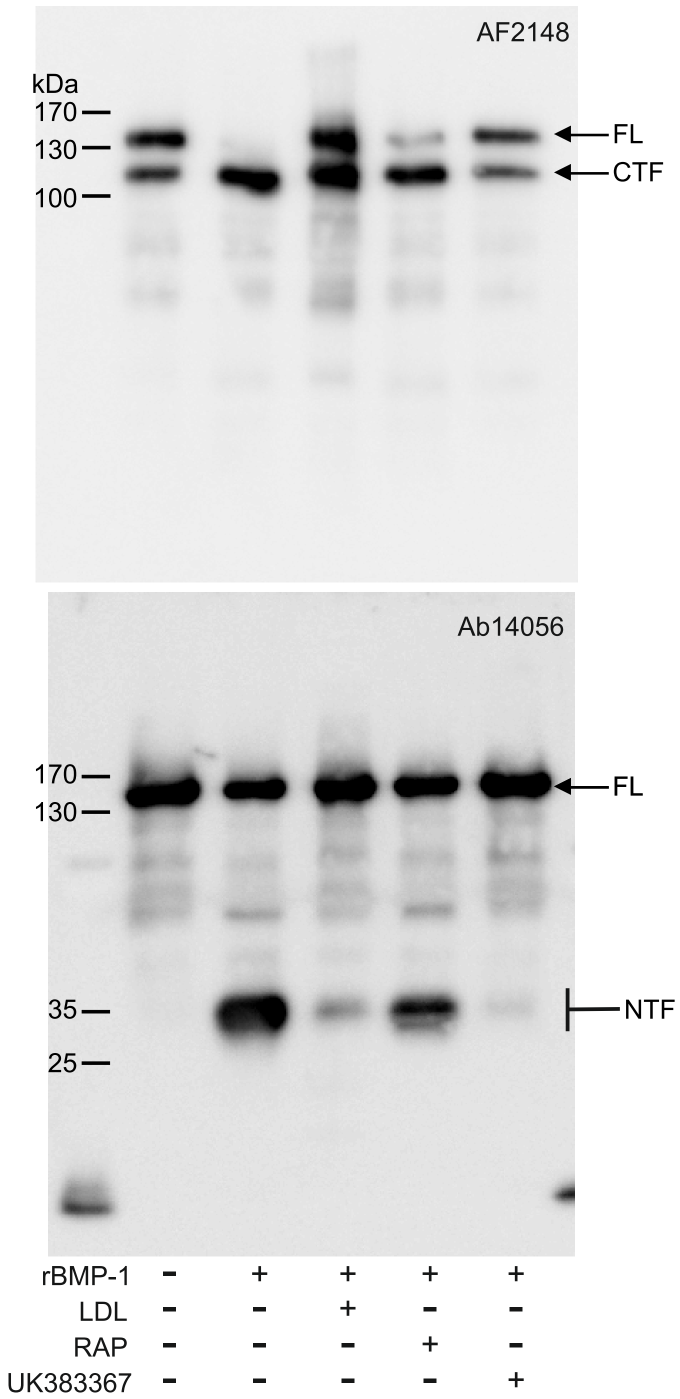

Figure 2A

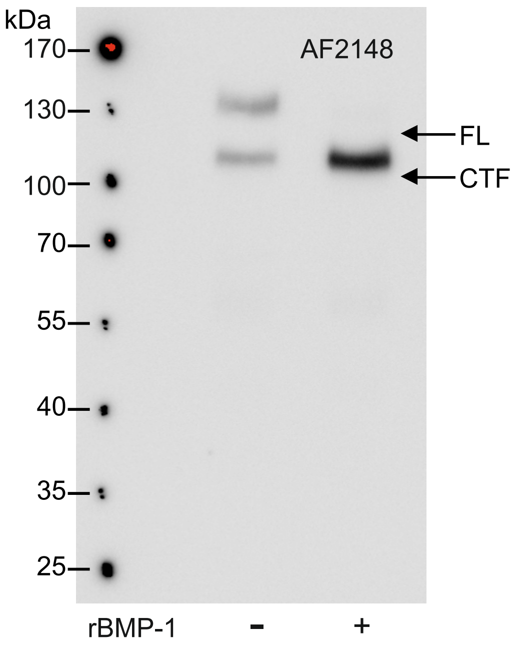

Figure 2E

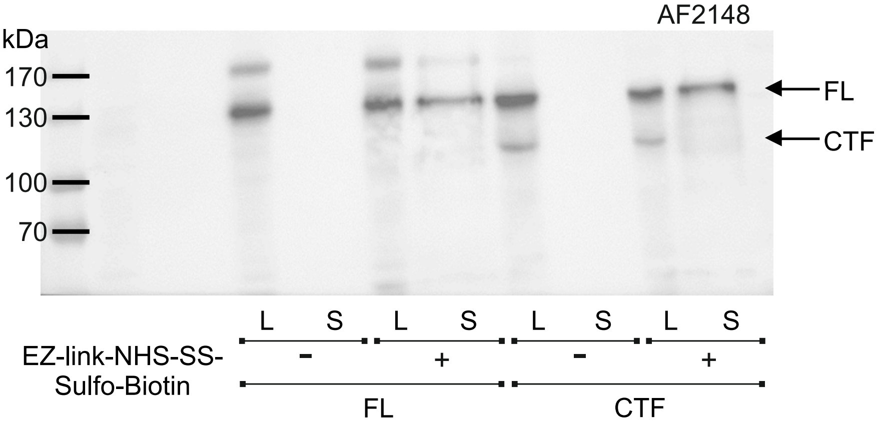

Figure 2D

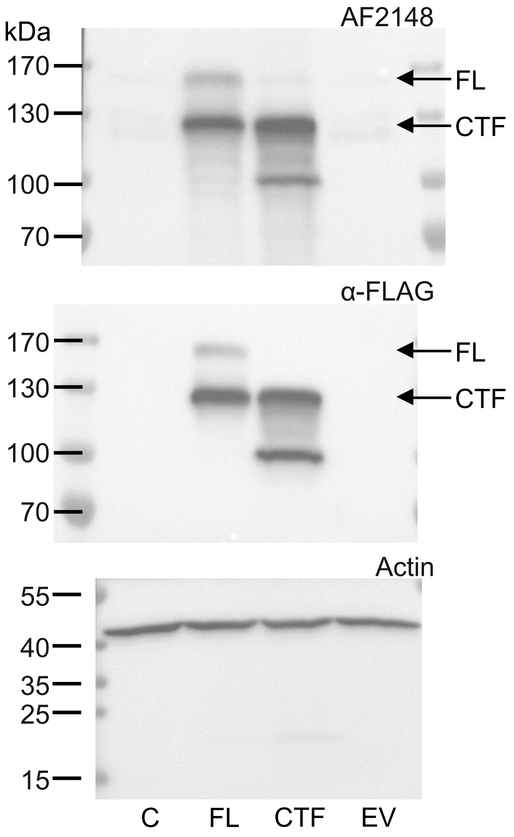

Figure 3A

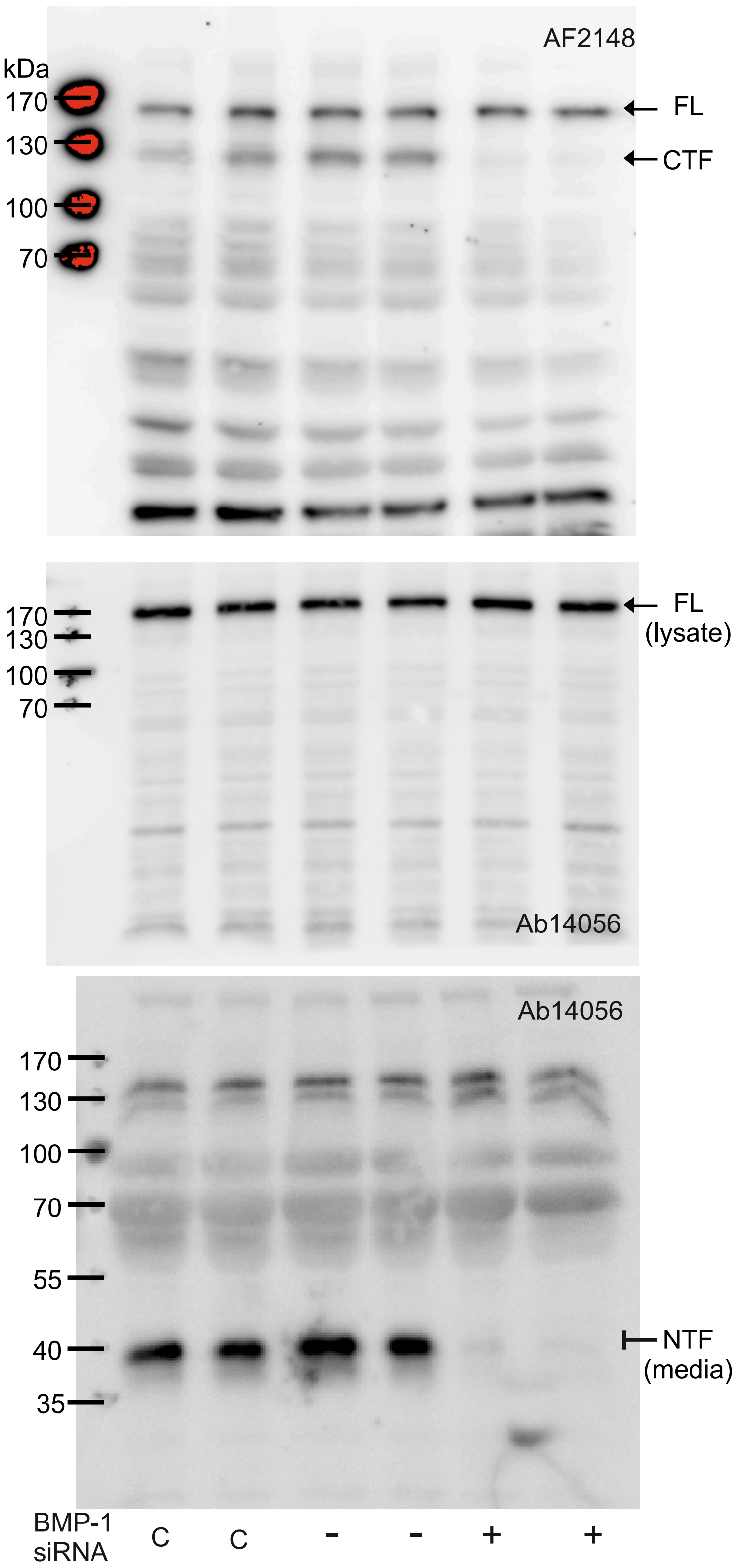

Figure 3C

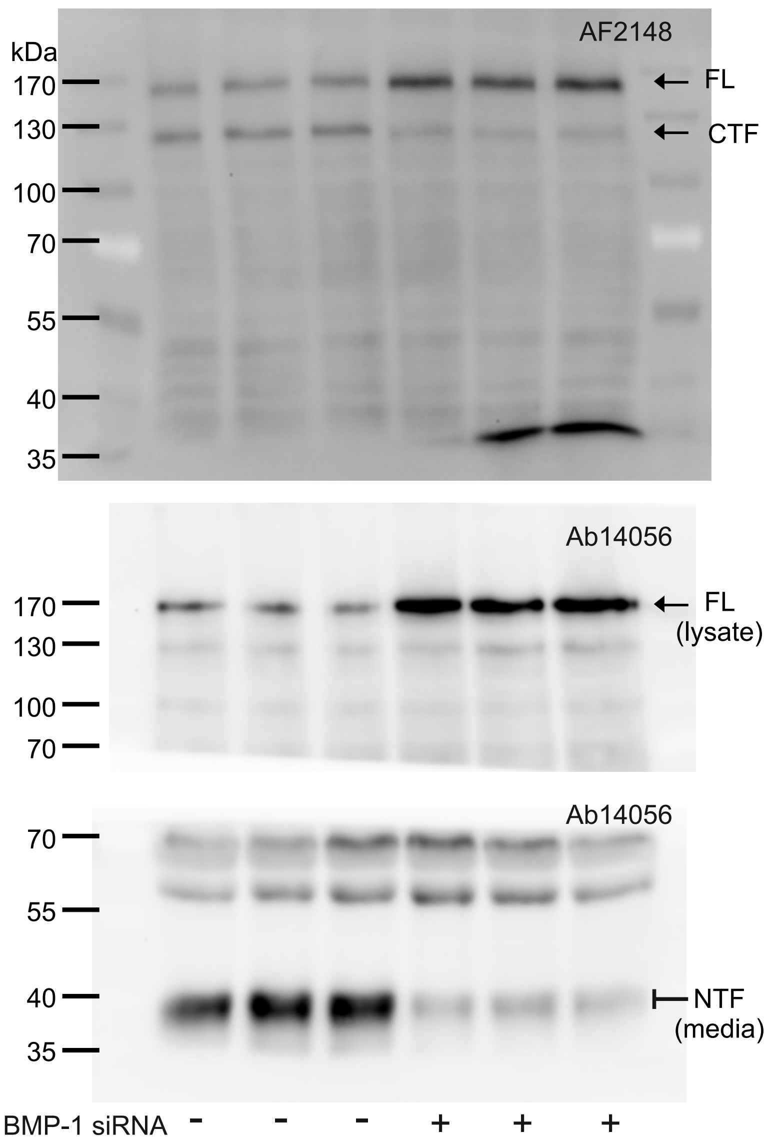

Figure 3E

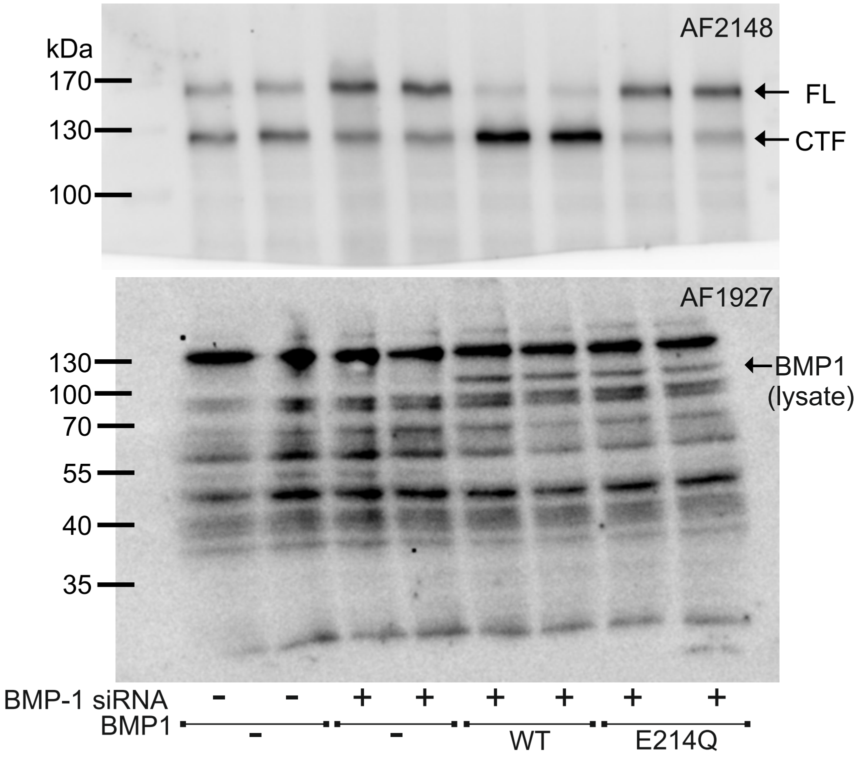

Figure 4A

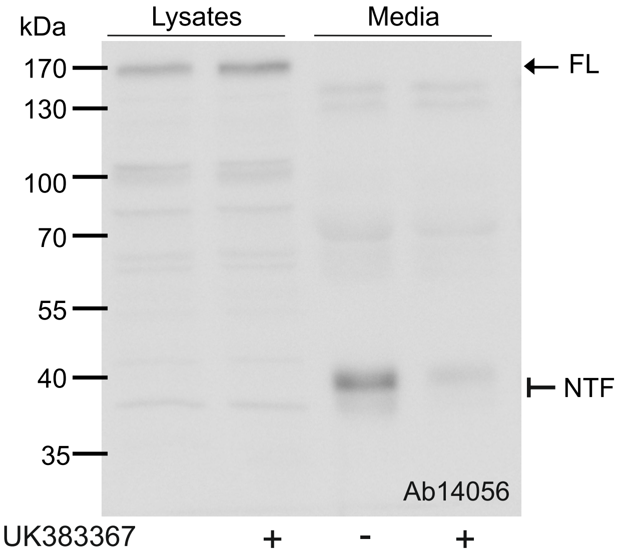

Figure 5A

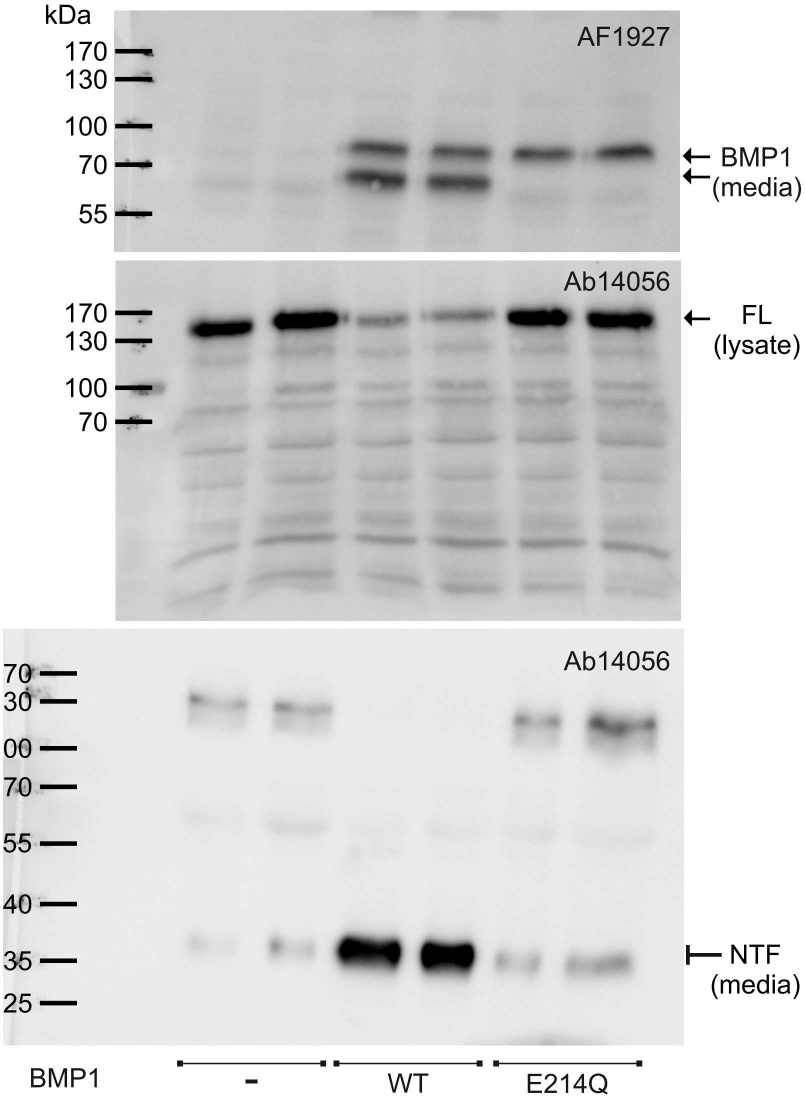

Figure 5G

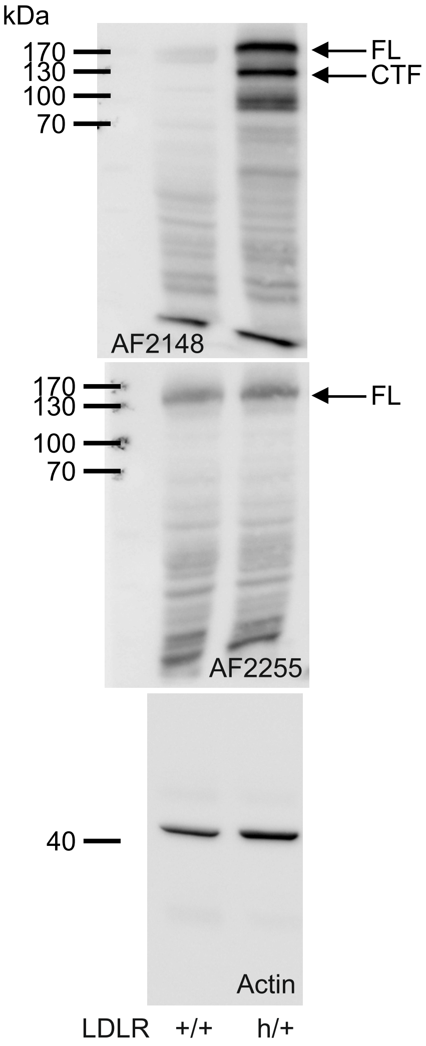

Figure 5E

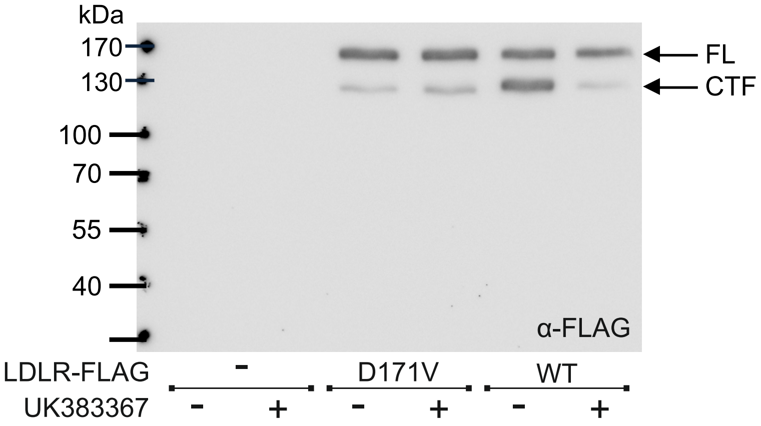

Supplement: Supplementary file 1 — Supplementary figures [file 41598_2019_47814_MOESM1_ESM.pdf]
